# Identification of the adhesive domain of AtaA from *Acinetobacter* sp. Tol 5 and its application in immobilizing *Escherichia coli*

**DOI:** 10.3389/fbioe.2022.1095057

**Published:** 2023-01-09

**Authors:** Shogo Yoshimoto, Sota Aoki, Yuki Ohara, Masahito Ishikawa, Atsuo Suzuki, Dirk Linke, Andrei N. Lupas, Katsutoshi Hori

**Affiliations:** ^1^ Department of Biomolecular Engineering, Graduate School of Engineering, Nagoya University, Nagoya, Japan; ^2^ Department of Biosciences, University of Oslo, Oslo, Norway; ^3^ Department of Protein Evolution, Max Planck Institute for Biology, Tübingen, Germany

**Keywords:** adhesion, immobilization, bacteria, protein engineering, autotransporter

## Abstract

Cell immobilization is an important technique for efficiently utilizing whole-cell biocatalysts. We previously invented a method for bacterial cell immobilization using AtaA, a trimeric autotransporter adhesin from the highly sticky bacterium *Acinetobacter* sp. Tol 5. However, except for *Acinetobacter* species, only one bacterium has been successfully immobilized using AtaA. This is probably because the heterologous expression of large AtaA (1 MDa), that is a homotrimer of polypeptide chains composed of 3,630 amino acids, is difficult. In this study, we identified the adhesive domain of AtaA and constructed a miniaturized AtaA (mini-AtaA) to improve the heterologous expression of *ataA.* In-frame deletion mutants were used to perform functional mapping, revealing that the N-terminal head domain is essential for the adhesive feature of AtaA. The mini-AtaA, which contains a homotrimer of polypeptide chains from 775 amino acids and lacks the unnecessary part for its adhesion, was properly expressed in *E. coli,* and a larger amount of molecules was displayed on the cell surface than that of full-length AtaA (FL-AtaA). The immobilization ratio of *E. coli* cells expressing mini-AtaA on a polyurethane foam support was significantly higher compared to the cells with or without FL-AtaA expression, respectively. The expression of mini-AtaA in *E. coli* had little effect on the cell growth and the activity of another enzyme reflecting the production level, and the immobilized *E. coli* cells could be used for repetitive enzymatic reactions as a whole-cell catalyst

## Introduction

Microbial cells are expected to provide environmentally friendly production processes as whole-cell biocatalysts with highly effective and selective reactivity under ordinary temperature and atmospheric pressure (Schmid et al., 2001; Pollard and Woodley, 2007; Fukuda et al., 2008; Taher and Chandran, 2013). Cell immobilization is an important strategy to efficiently use fragile whole-cell biocatalysts because it simplifies product separation, enables the cell concentration to be increased, confers tolerance to toxic substances, and permits repetitive or continuous use of precious and expensive biocatalysts (Hartmeier, 1985; Junter and Jouenne, 2004; Lee and Kim, 2015; Lotti et al., 2018). Conventional methods for cell immobilization are gel entrapment, covalent bonding to solid surfaces, cross-linking, and physical adsorption (Klein and Ziehr, 1990; Smidsrod and Skjakbraek, 1990). However, these methods have practical limitations, such as limited mass transfer in the inner part of a gel (Cassidy et al., 1996; Carballeira et al., 2009), gel fragility, cell leakage from the support matrix, and adverse effects on cell viability and catalytic activity (Junter and Jouenne, 2004). Biofilms, which are composed of cells and extracellular polymeric substances (EPS), are also utilized in biofilm reactors (Qureshi et al., 2005; Halan et al., 2012), but their use is limited because the immobilization efficiency depends on the culture conditions and ability of each bacterial strain to form biofilms. Therefore, new immobilization technologies that can be easily and widely used are desired.

The Gram-negative bacterium *Acinetobacter* sp. Tol 5 shows extremely high adhesiveness to various material surfaces, including hydrophobic plastics, hydrophilic glass and metals, independent of cell growth and of the secretion of EPS (Ishikawa et al., 2012; Furuichi et al., 2020). The adhesiveness of Tol 5 is attributed to its fibrous cell surface protein, AtaA (Ishikawa et al., 2012; Ishii et al., 2022). AtaA is a member of the trimeric autotransporter adhesin (TAA) family (Linke et al., 2006), and polypeptide chains of AtaA consisting of 3,630 amino acids form a very large homotrimeric fibrous structure that is over 1 MDa in molecular weight and 260 nm in length. This large AtaA protein is composed of a passenger domain (PSD) and the C-terminal transmembrane domain (TM) that anchors in the outer membrane (OM). The PSD consists of an N-terminal head domain (Nhead), multiple types of domain repeats, and coiled-coil connectors (Bassler et al., 2015). While AtaA shares structural features with other TAAs, which usually bind bacterial cells to specific biotic molecules (Meuskens et al., 2019), AtaA is unique as it exhibits non-specific, high adhesiveness to various abiotic surfaces. In addition, this adhesive property can be conferred to other bacterial cells by heterologous expression of the *ataA* gene.

Utilizing AtaA, we have developed a new method for bacterial cell immobilization (Ishikawa et al., 2014). Its advantages over conventional methods are that any material can be selected as the immobilization support, it is rapid and simple, and there is no mass transfer limitation. The greatest advantage is reversible cell immobilization and removal, which allows for reuse of both the cells and the support. Bacterial cells can be immobilized directly on the surface of the support within minutes of contact with the support in suspension without the EPS production. Its adhesiveness is so strong that the cells do not detach easily during agitation or flow in the bioprocess. However, since it is due to physical adsorption *via* AtaA, the reduction of cell activity due to chemical bonding can be avoided (Ishikawa et al., 2014; Hori et al., 2015). Nevertheless, the cells can be detached in deionized water or a casamino acid solution, and yet can be re-immobilized in normal buffer solution (Yoshimoto et al., 2017; Ohara et al., 2019). However, there are disadvantages in this new immobilization method. It is, in principle, applicable only to Gram-negative bacteria. Heterologous expression of the large protein AtaA is not easy, even in Gram-negative bacteria (Rosano and Ceccarelli, 2014). It is thought that producing such a huge foreign protein in such large quantities that it covers the cell would impose a large energy burden on the host. In fact, except for *Acinetobacter* species, only one bacterium, *Enterobacter aerogenes*, has been efficiently immobilized using AtaA so far (Nakatani et al., 2018; Hori et al., 2022). In the case of *Escherichia coli*, an important model and industrial bacterium (Huffer et al., 2012; Pontrelli et al., 2018; Thomson et al., 2018; Gleizer et al., 2019; Yang et al., 2020; Liu et al., 2021; Zhang et al., 2021; Rinaldi et al., 2022), the amount of AtaA displayed on the cell surface was very low (Nakatani et al., 2019). In this study, we aimed to identify the adhesive AtaA domain that is responsible for the strong adhesion to surfaces by functional mapping and to construct a miniaturized AtaA for the effective immobilization of *E. coli* cells through improving the heterologous expression of *ataA*.

## Materials and methods

### Bacterial strains and culture conditions

The bacterial strains used in this study are listed in Table 1. These bacterial strains were grown as described previously (Ishikawa et al., 2012). *Acinetobacter* sp. Tol 5 and its mutant strains were grown at 28°C and *E. coli* strains were grown at 37°C in lysogeny broth (LB) medium unless otherwise noted. The following antibiotics were used at the following concentrations when necessary: ampicillin (500 μg/mL) and gentamicin (10 μg/mL) for Tol 5 mutant strains and ampicillin (100 μg/mL), gentamicin (10 μg/mL) and kanamycin (50 μg/mL) for *E. coli*. For the expression of *ataA* and its mutants, an overnight culture of bacterial cells harboring the expression vector was inoculated into LB medium supplemented with ampicillin, gentamicin, and 0.5% (w/v) arabinose to induce the expression of genes under the control of the P_BAD_ promoter, and the medium was incubated for 7 h at 115 rpm. Growth curves were obtained by measuring the optical density at 600 nm (OD_600_) of the culture medium.

**TABLE 1:** Bacterial strains used in this study.

Strain	Description	Reference
*Acinetobacter* sp. Tol 5	Wild type strain	Hori et al. (2001)
*Acinetobacter* sp. Tol 5 4140	Unmarked Δ*ataA* mutant of *Acinetobacter* sp. Tol 5, *ataA* ^*-*^	Ishikawa and Hori (2013)
*E. coli* DH5α	Host for routine cloning	Purchased from Takara (Shiga, Japan)
*E. coli* BL21 (DE3)	Host for protein expression and immobilization assay	Purchased from Thermo Fisher Scientific (Waltham, MA, United States)
*E. coli* JCM-Tfu0937 (bgl)-Blc	JCM20137; HCEpromoter, Tfu0937-Blc, and terminator region are integrated into its genome, host for repetitive enzymatic reaction	Tanaka et al. (2014)
*E. coli* JCM (*bgl*)	*E. coli* JCM-Tfu0937 (bgl)-Blc transformed with pARP3	This study
*E. coli* JCM (*bgl*, *mini-ataA*)	*E. coli* JCM-Tfu0937 (bgl)-Blc transformed with pIFD-mini-AtaA	This study

### Construction of plasmids

The plasmids and primers used in this study and synthetic DNA fragments prepared in this study are listed in Table 2, Supplementary Tables S1, S2, respectively. The schematic procedures for the construction of *ataA* mutants in pDONR221 plasmids are shown in Supplementary Figures S1–S7. The details of the procedure are described below.

**TABLE 2:** Plasmids used in this study.

Name	Description	Reference
pARP3	Expression vector for broad host, Amp^r^, Gm^r^, *araC*-P_BAD_	Ishikawa et al. (2012)
pDONR221	Cloning vector, Km^r^	Purchased from Thermo Fisher Scientific
pTA2	Cloning vector for the TA cloning system, Amp^r^	Purchased from Toyobo
pMD19	Cloning vector, Amp^r^	Purchased from Takara
pTAKN-2	Cloning vector, Km^r^	Purchased from Eurofins (Luxembourg)
pAtaA	The expression vector encoding full length *ataA*, pARP3:ataA, Amp^r^, Gm^r^	Ishikawa et al. (2012)
pDONR221:*ataA*	The vector encoding full length *ataA*, Km^r^	Ishikawa et al. (2012)
pDONR221:*Ntemp*	The vector encoding *Ntemp* (AtaA_1-496_), Km^r^	Aoki et al. (2020)
pIFD-∆Nhead	pARP3 vectors encoding *ataA* fragment carrying an in-frame deletion of 60–313 aa	This study
pIFD-∆NS-A1	pARP3 vector encoding *ataA* fragment carrying an in-frame deletion of 327–506 aa	This study
pIFD-∆NS-A2	pARP3 vector encoding *ataA* fragment carrying an in-frame deletion of 507–1337 aa	This study
pIFD-∆NS-B	pARP3 vector encoding *ataA* fragment carrying an in-frame deletion of 1338–2335 aa	This study
pIFD-∆NS-C∆Chead	pARP3 vector encoding *ataA* fragment carrying an in-frame deletion of 2397–3169 aa	This study
pIFD-∆Cstalk	pARP3 vector encoding *ataA* fragment carrying an in-frame deletion of 3170–3475 aa	This study
pIFD-mini-AtaA	pARP3 vector encoding *ataA* fragment carrying an in-frame deletion of 364–3218 aa	This study

To construct pIFD-∆Nhead, a DNA fragment was amplified by inverse PCR (iPCR) from pDONR221:*Ntemp* using primers ∆Nhead-f/∆Nhead-r and self-ligated, generating pDONR221:*Ntemp_∆Nhead*. A DNA fragment excised from pDONR221:*ataA* with EcoRV was inserted into the same site of pDONR221:*Ntemp_∆Nhead*, generating pDONR221:*∆Nhead*.

To construct pIFD-∆NS-A1, pTAKN-2:*FragTrp3,4* was digested with BsaI, and the resulting fragment was inserted into the same site of pTAKN-2:*FragTrp5,6,* generating pTAKN-2:*FragTrp3-6*. A DNA fragment excised from pMD19:*FragC* with BglII/BamHI was inserted into the BamHI site of pMD19:*FragB*, generating pMD19:*FragBC*. Then, a DNA fragment was amplified by iPCR from pDONR221:*FragA2_031* using primers ∆NS-A1-f1/∆NS-A1-r1 and self-ligated, generating pDONR221:*FragA2_054*. A DNA fragment excised from pTAKN-2:*FragTrp3-6* with BsaI/BamHI was inserted into the same site of pDONR221:*FragA2_054,* generating pDONR221:*FragA_054*. A DNA fragment excised from pMD19:*FragBC* with BglII/BamHI was inserted into the BamHI site of pDONR221:*pFragA_054*, generating pDONR221:*∆NS-A1*.

To construct pIFD-ΔNS-A2, a DNA fragment was amplified by PCR from pDONR221:*ataA* using primers ∆NS-A2-f1/∆NS-A2-r1 and self-ligated, generating pDONR221:*∆Nstalk-a*. A DNA fragment was amplified by PCR from pMD19:*FragB* using primers ∆NS-A2-f2/∆NS-A2-r2 and assembled with the longer DNA fragment of pMD19:*FragB* digested with BglII/PstI using In-Fusion HD Cloning Kit (Takara Bio, Shiga Japan), generating pMD19:*FragB_09.* A DNA fragment excised from pMD19:*FragC* with BglII/BamHI was inserted into the BamHI site of pMD19:*FragB_09,* generating pMD19:*FragBC_09.* Then, a DNA fragment excised from pMD19:*FragBC_09* with BsaI/BamHI was inserted into the same site of pDONR221:*∆Nstalk-a*, generating pDONR221:*∆NS-A2.*

To construct pIFD-ΔNS-B, a DNA fragment was amplified by PCR from pDONR221:*ataA* using primers ∆NS-B-f1/∆NS-B-r1 and was self-ligated, generating pDONR221:*∆Nstalk-b*. A DNA fragment was amplified by PCR from pMD19:*FragC* using primers ∆NS-B-f2/∆NS-B-r2 and was assembled with the pTA2 vector (TArget Clone; TOYOBO, Osaka, Japan), generating pTA2:*FragC_13.* A DNA fragment excised from pTA2:*FragC_13* with BsaI/BamHI was inserted into the same site of pMD19:*FragA_013.* The resulting plasmid was digested with BglII/BamHI and inserted into the BamHI site of pDONR221:*∆Nstalk-b*, generating pDONR: *ΔNS-B.*

To construct pIFD-∆NS-C∆Chead, a DNA fragment was amplified by iPCR from pDONR221:*∆NS-A2* using primers ∆NS-C∆Chead-f/∆NS-C∆Chead-r and was self-ligated, generating pDONR221:*∆NS-A2a*. Subsequently, a DNA fragment excised from pTAKN-2:Trp11_*047* *&* *048* with BglII/BamHI was inserted into the BamHI site of pMD19:*FragB*. The resulting plasmid was digested with BglII/BamHI and inserted into the BamHI site of pMD19:*FragA*. Then, the resulting plasmid was digested with BglII/BsaI and inserted into the BamHI/BsaI sites of pDONR221:*∆NS-A2a*, generating pDONR221:*∆NS-C∆Chead.*

To construct pIFD-∆Cstalk, a DNA fragment excised from pDONR221:*ataA* with KpnI/XbaI was inserted into the same site of the pTA2 vector, generating pTA2:*Ctemp*. A DNA fragment was amplified by iPCR from pTA2:*Ctemp* using primers ∆Cstalk-f/∆Cstalk-r and was self-ligated*.* The resulting plasmid was digested with BglII/XbaI and inserted into the same site of pDONR221:*ataA*, generating pDONR221:*∆Cstalk*.

To construct pIFD-mini-AtaA, a DNA fragment was amplified by iPCR from pDONR221:*ataA* using primers mini-AtaA-f/mini-AtaA-r and was self-ligated, generating pDONR221:*mini-ataA*.

Finally, all of these *ataA* mutants in pDONR221 plasmids were excised with EcoRI/XbaI and inserted into the same site of pARP3, generating plasmids for expression. Transformation of the Δ*ataA* mutant strain Tol 5 4140 with these expression vectors was carried out by conjugal transfer from the *E. coli* S17-1 strain as previously described (Ishikawa et al., 2012).

### Protein detection

The expression and production of mutant variants of AtaA were detected by SDS–PAGE followed by Coomassie brilliant blue (CBB) staining and immunoblotting as described previously (Ishikawa et al., 2012) with a slight modification. To confirm the production of AtaA and its mutants in *E. coli*, SDS-sample buffer (5% (v/v) 2-mercaptoethanol, 2% (w/v) SDS, 0.02% (w/v) bromophenol blue, 62.5 mM Tris-HCl, pH 6.8) supplemented with 8 M urea and anti-AtaA_59-325_ rabbit antiserum (Aoki et al., 2020) was used.

### Flow cytometry

Flow cytometry was performed as described previously (Ishikawa et al., 2012) with a slight modification. In brief, bacterial cells were resuspended in PBS containing 4% (w/v) paraformaldehyde and incubated at room temperature for 15 min. The samples were washed with PBS and treated with anti-AtaA_699-1014_ (Ishikawa et al., 2012) or anti-AtaA_59-325_ (Aoki et al., 2020) rabbit antiserum at a 1:10000 dilution in PBS containing 0.05% (v/v) Tween 20. After a 30-min incubation at room temperature, the samples were washed twice with NET buffer (150 mM NaCl, 5 mM EDTA, 50 mM Tris-HCl, 0.05% (v/v) Triton X-100, pH 7.6) and treated with Alexa Fluor 488-conjugated anti-rabbit antibody (Cell Signaling Technology, MA) at a 1:500 dilution in NET buffer for 30 min. The samples were washed twice with NET buffer and resuspended in deionized water, and the fluorescence was measured by FACS Canto II (Becton, Dickinson and Company, NJ, United States). Histograms were created using Flowjo software (Tomy Digital Biology, Tokyo, Japan).

### Immunofluorescence microscopy

Immunofluorescence microscopy was performed as described previously (Ishikawa et al., 2016) with a slight modification. In brief, bacterial cells were placed onto a gelatin-coated glass plate, fixed with PBS containing 4% (w/v) paraformaldehyde solution for 15 min, and washed twice with PBS. Anti-AtaA_59-325_ rabbit antiserum at a 1:10,000 dilution in PBS containing 0.05% (v/v) Tween 20, was placed onto the sample plate. After a 30-min incubation, the sample plate was washed with NET buffer, incubated for 30 min with Alexa Fluor 488-conjugated anti-rabbit antibody at a 1:500 dilution in NET buffer. The sample plate was washed twice with PBS and observed under a confocal laser-scanning microscope (FV-1000, Olympus Corporation, Tokyo, Japan).

### Adhesion assay using microwell plates

Adhesion assays using microwell plates were performed as previously described (Ishikawa et al., 2012). In brief, 200 μl of a bacterial cell suspension in BS-N buffer (34.5 mM Na_2_HPO_4_, 14.7 mM KH_2_PO_4_, 15.5 mM K_2_SO_4_, pH 7.2) at an optical density at 660 nm of 0.5 was placed into a 96-well PS plate (353072; Becton, Dickinson and Company, NJ, United States) or a 96-well glass plate (FB-96; Nippon Sheet Glass Co., Ltd., Tokyo, Japan). After incubation for 2 h at 28°C without shaking, the cell suspensions were removed by a micropipette, and each well was rinsed three times with 200 μl of BS-N buffer. Cells adhering to the microwell surface were stained with 0.1% (w/v) crystal violet solution for 15 min. After three rinses with 200 μl of BS-N buffer, the stain was eluted from the cells with 200 μl of 70% (v/v) ethanol, and the absorbance at 590 nm of the elution was measured by a microplate reader (ARVO X3; PerkinElmer, MA, United States).

### Immobilization of bacterial cells on polyurethane foam supports

The immobilization of bacterial cells on polyurethane foam supports was performed as described previously (Yoshimoto et al., 2017), with slight modifications. The bacterial cells were suspended in 30 ml of BS-N buffer at an OD_600_ of 1.0 in a 100-mL Erlenmeyer flask. Five pieces of polyurethane foam support with a specific surface area of 50 cm^2^/cm^3^ (CFH-40; Inoac Corporation, Nagoya, Japan) in the shape of a cube (1 cm^3^) were placed into the cell suspension and shaken at 115 rpm at 28°C for 60 min. After shaking, the OD_600_ of the cell suspension was measured. The immobilization ratio of the cells was calculated using the following equation:

$$Immobilization\;ratio\left(\%\right)=100\times \left({OD}_{600\;initial}-{OD}_{600\;after\;shaking}\right)/{OD}_{600\;initial}$$ (1)

For the detachment assay, the support with immobilized cells was placed into a 100-mL Erlenmeyer flask containing 25 mL of BS-N buffer supplemented with or without 1% (wt/vol) casein hydrolysate (casamino acids technical grade; Becton, Dickinson and company) and shaken at 115 rpm at 28°C. After 5 min of shaking, the OD_600_ of the suspension containing detached cells was measured (OD_600_-detached). The detachment ratio of the cells was calculated from the following equation:

$$Detachment\;ratio\left(\%\right)=100\times {OD}_{600\;detached}/\left({OD}_{600\;initial}-{OD}_{600\;after\;shaking}\right)$$ (2)

For the re-immobilization assay, the cell suspension containing detached cells and 1% casein hydrolysate was centrifuged (5,000×g, 10 min, 25°C), and the precipitated cells were rinsed with BS-N buffer three times. The cells were resuspended in BS-N buffer at an OD_600_ of 1.0 and shaken with 5 pieces of polyurethane foam support in a 100-mL Erlenmeyer flask at 115 rpm at 28°C. The OD_600_ of the cell suspension was measured periodically, and the immobilization ratio was calculated from Equation 1.

### Microscopic observation of polyurethane support

The polyurethane support on which the bacterial cells were immobilized was rinsed in BS-N buffer. The support was sliced into 1 mm-thick and immersed in 4% paraformaldehyde phosphate buffer solution (FUJIFILM Wako, Osaka, Japan) for 15 min. After a rinse in BS-N buffer, the immobilized cells on the support were stained with 60 µM propidium iodide (Invitrogen, Waltham) dissolved in 50 mM HEPES buffer (pH 7.4) for 15 min. The support was rinsed in BS-N buffer and observed in BS-N buffer under a digital microscope (KEYENCE, Osaka, Japan) at ×100 magnification.

### Enzymatic reaction

For reactions in cell suspension, cells of *E. coli* JCM-Tfu0937 (bgl)-Blc derivatives expressing β-glucosidase (BGL) constitutively were suspended in 10 ml of BS-N buffer in a 15-mL centrifuge tube to an OD_600_ of 1.0. The reaction was initiated by adding 4-nitrophenyl β-D-glucopyranoside (FUJIFILM Wako) at a final concentration of 1 mM, and the absorbance at 405 nm was measured after shaking at 37°C for 30 min. The concentration of *p*-nitrophenol produced by BGL was calculated from the increase in absorbance at 405 nm.

For reactions with immobilized cells, the polyurethane support on which cells of *E. coli* JCM (*bgl*, *mini-ataA*) were immobilized was rinsed in BS-N buffer and dewatered on paper towel for 2 min. One piece of the support with the immobilized cells were incubated in 5 ml of BS-N buffer containing 0.2 mM 4-nitrophenyl β-D-glucopyranoside at 37°C with shaking and the absorbance of at 405 nm was measured periodically. After 50-min reaction, the support was collected, washed with fresh BS-N buffer, transferred to a fresh reaction solution, and subjected to a new cycle of the reaction.

## Results

### Construction and expression of IFD mutant genes of AtaA

**FIGURE 1:**
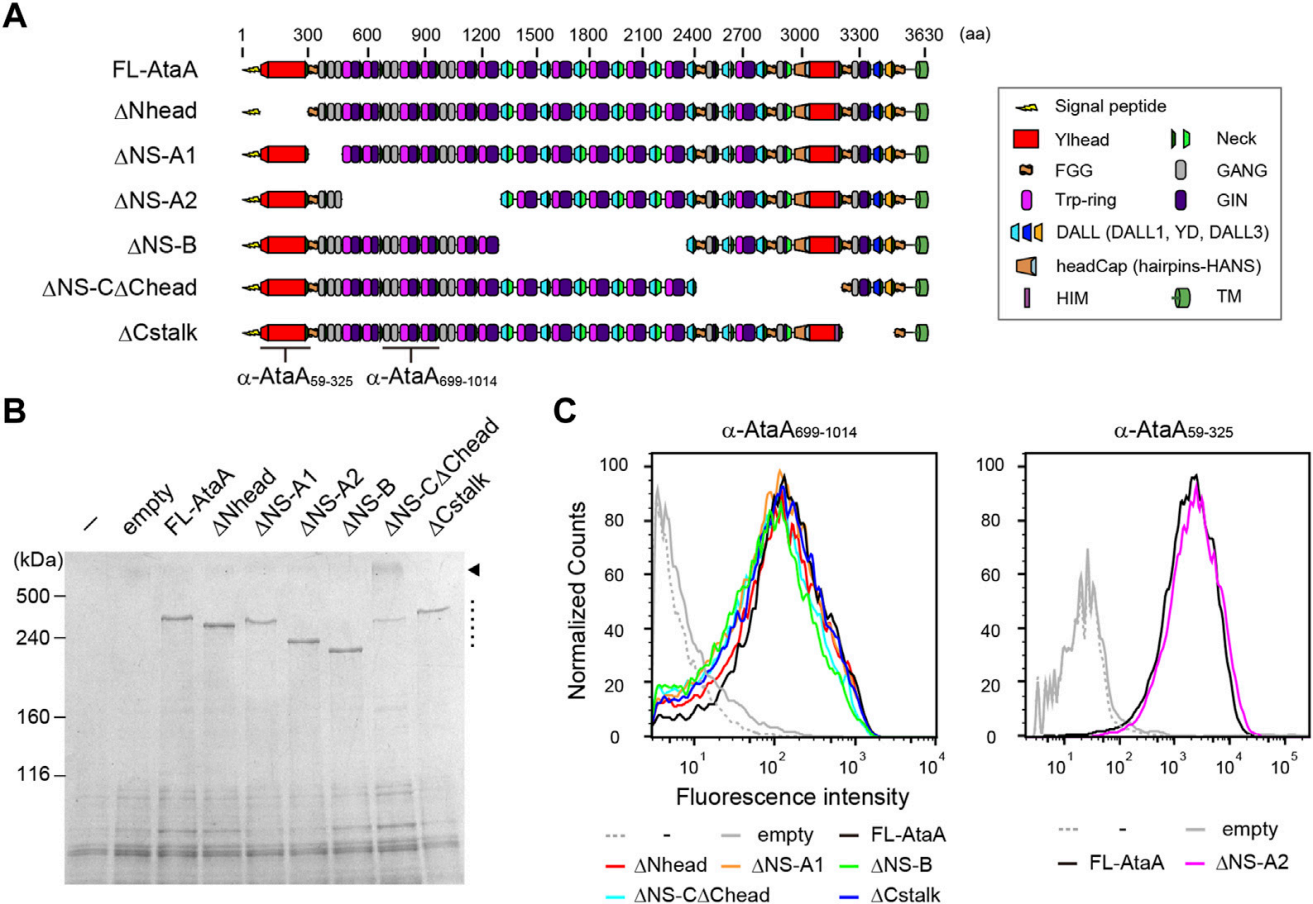
Functional mapping of AtaA by in-frame deletion (IFD). **(A)** Schematic representation of IFD mutant proteins of AtaA: IFD-ΔNhead (Δ60-313 aa), ΔNS-A1 (Δ327–506 aa), ΔNS-A2 (Δ507–1337 aa), ΔNS-B (Δ1338–2335 aa), ΔNS-CΔChead (Δ2397–3169 aa), and ΔCstalk (Δ3177–3475 aa). **(B)** SDS–PAGE followed by CBB staining of whole-cell lysates from Tol 5 Δ*ataA* cells expressing FL-AtaA or IFD mutant genes. For the controls, Tol 5 Δ*ataA* (−) and Tol 5 Δ*ataA* harboring the empty vector were also subjected to the same analyses. A dotted line and an arrowhead indicate the bands corresponding to the monomer of IFD mutant proteins and the trimer of IFD-ΔNS-CΔChead, respectively. **(C)** Flow cytometry of the Tol 5 Δ*ataA* cells expressing FL-AtaA or IFD mutant genes using anti-AtaA_699-1014_ antiserum or anti-AtaA_59-325_ antiserum. Note that the two antisera do not react with all constructs. The positions for recognition of the antisera are indicated in panel **(A)**.

To evaluate the contribution of each domain of AtaA to the adhesiveness, we constructed six in-frame deletion (IFD) mutant genes for plasmid-based expression in Δ*ataA* strain of *Acinetobacter* sp. Tol 5, as shown in Figure 1A. When constructing domain-deletion mutant genes of large and complex proteins, great care and precise design are necessary to prevent undesirable structural distortion and steric conflicts in the resulting mutant proteins. Recently, we confirmed that preserving the periodicity of hydrophobic residues in coiled coils at the deletion region is effective for displaying the very large homotrimeric fiber structure and maintaining the proper folding state of AtaA on the cell surface after a domain is deleted (Aoki et al., 2020). Therefore, each IFD mutant protein was carefully and precisely designed and constructed so that coiled-coil periodicity was maintained after deletion, following established procedures for TAA domain prediction (Szczesny and Lupas, 2008; Bassler et al., 2015). The IFD mutant genes were expressed in the Tol 5 Δ*ataA* strain Tol 5 4140, and successful recombinant protein production was confirmed by SDS–PAGE followed by CBB staining. All of the IFD mutant proteins were detected as well as full-length AtaA (FL-AtaA) (Figure 1B, dotted line). A band corresponding to a higher molecular weight was also detected in IFD-ΔNS-CΔChead (Figure 1B, arrowhead). The band was considered to be an undenatured trimeric state of the mutant protein because many TAAs are known to have a tolerance to heat denaturation (Grosskinsky et al., 2007; Riess et al., 2007) that is not easily resolved by excess heating. Subsequently, we examined the cell surface display of the IFD mutant proteins by flow cytometry. Tol 5 4140 cells expressing the IFD mutant genes except IFD-ΔNS-A2 were immunostained with anti-AtaA_699-1014_ antiserum and Alexa 488-conjugated anti-rabbit antibody. For IFD-ΔNS-A2, which lacks the antigenic region relevant for the anti-AtaA_699-1014_ antiserum, anti-AtaA_59-325_ antiserum was used instead. All of the cells expressing the IFD mutant genes exhibited a high fluorescence intensity similar to that of the cells expressing FL-AtaA (Figure 1C). These results suggest that all of the IFD mutant proteins were properly produced, secreted, and displayed on the cell surface.

### Adhesive property of IFD mutant proteins of AtaA

**FIGURE 2:**
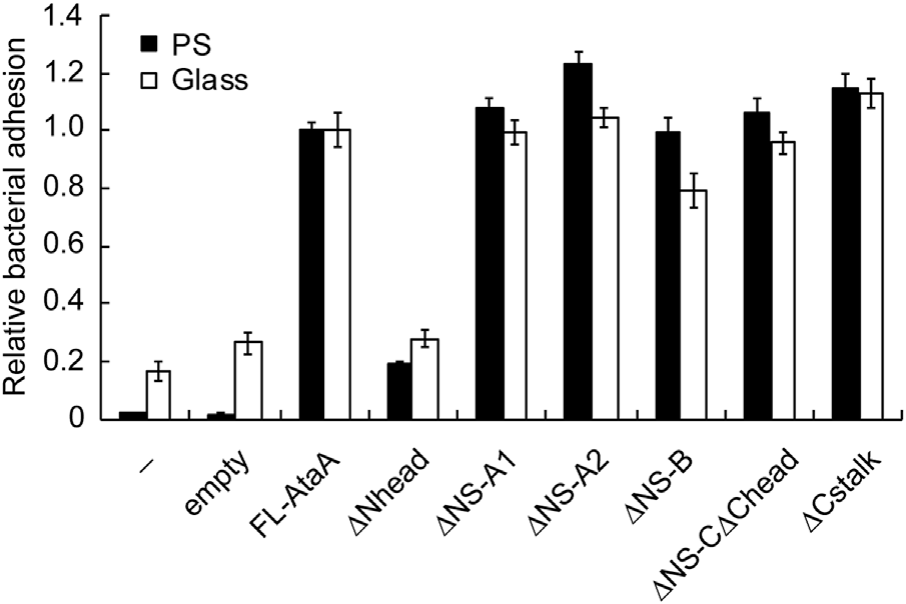
Adhesion assay of the Tol 5 Δ*ataA* cells expressing FL-AtaA or IFD mutants. The amount of adhered cells onto polystyrene (PS) and glass microwell plates was quantified by crystal violet staining and shown in relative values with FL-AtaA as the control. The data are represented as the mean ± SEM (n = 8). (−), Tol 5 Δ*ataA* cells without any plasmid; empty, Tol 5 Δ*ataA* cells harboring the empty vector.

Next, the adhesiveness of *Acinetobacter* cells expressing the IFD mutant proteins was evaluated by an adhesion assay using microwell plates. The cell suspensions of the Tol 5 4140 cells expressing the IFD mutant genes were placed into hydrophobic polystyrene (PS) and hydrophilic glass microwell plates. After an incubation for 2 h, the cell suspensions containing non-adhering cells were removed, and the adhered cells that remained in the microwell plates were quantified by crystal violet staining. The cells expressing the Nhead-deleted mutant (IFD-ΔNhead; Δ60-313) exhibited a significantly lower adhesiveness to both PS and glass surfaces than that of the cells expressing FL-AtaA (Figure 2), although a similar amount of AtaA molecules was displayed on the cell surface as described above (Figure 1C). In contrast, cells displaying other IFD mutant proteins maintained the same degree of high adhesiveness as that of FL-AtaA. Thus, Nhead was revealed to be the essential domain for the adhesive function of AtaA.

### Construction and expression of miniaturized AtaA in *E. coli*

**FIGURE 3:**
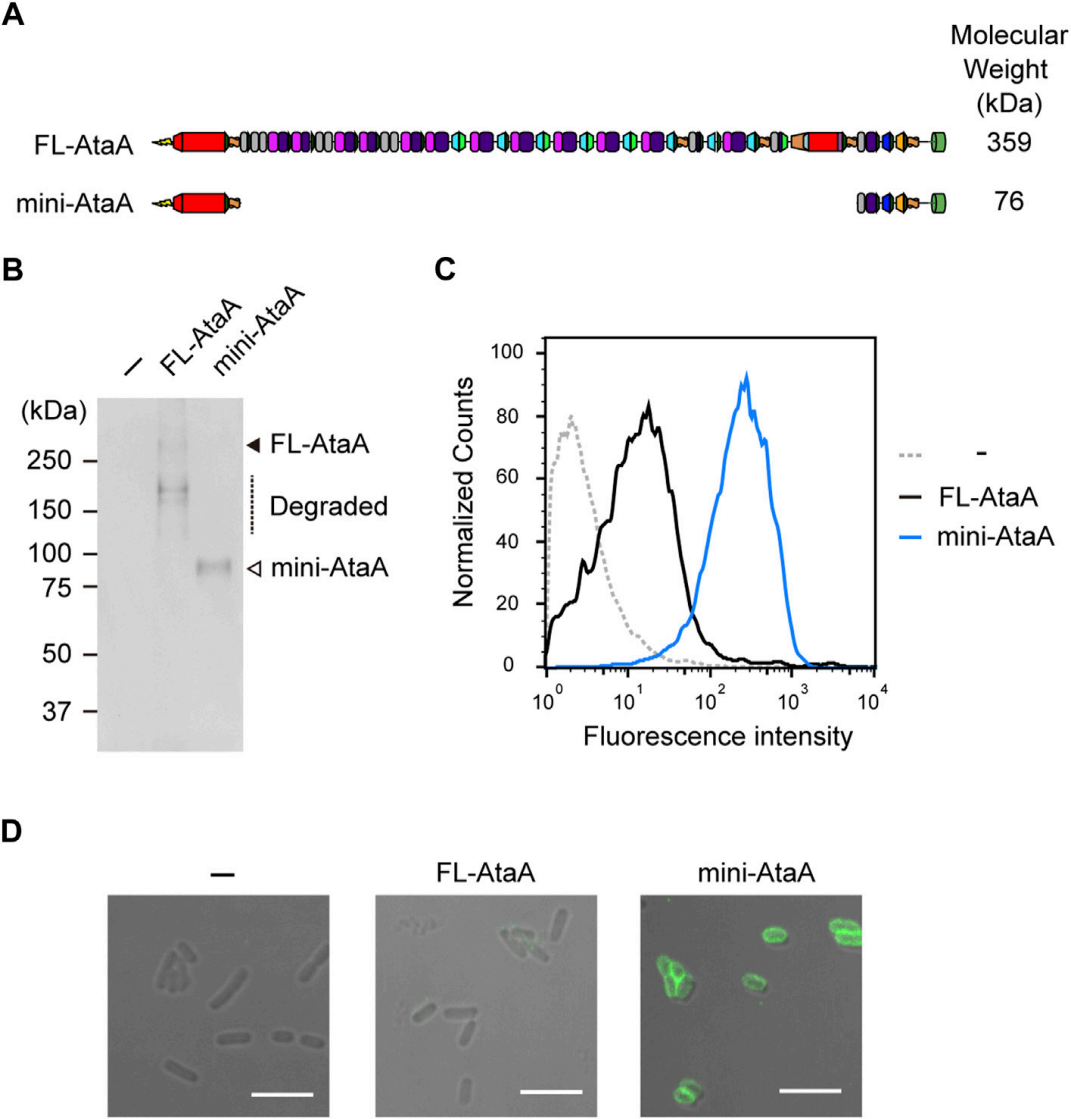
Expression of the miniaturized AtaA (mini-AtaA) in *E. coli*. **(A)** Schematic representation of the designed mini-AtaA. **(B)** Immunoblotting of the whole-cell lysates of *E. coli* WT (−), *E. coli* (*FL-ataA*), and *E. coli* (*mini-ataA*) using anti-AtaA_59-325_ antiserum. The black and white arrowheads and a dotted line indicate bands corresponding to FL-AtaA, mini-AtaA, and degraded FL-AtaA, respectively. **(C,D)** Flow cytometry **(C)** and immunofluorescence microscopy **(D)** of *E. coli* WT (−), *E. coli* (*FL-ataA*), and *E. coli* (*mini-ataA*) using anti-AtaA_59-325_ antiserum. Scale bar: 5 μm.

To extend the applicability of efficient heterologous expression of *ataA* to bacteria other than *Acinetobacter* species, we designed a miniaturized AtaA (mini-AtaA; Δ364-3218 aa). The mini-AtaA consists of a signal peptide (1-59 aa) for secretion through the inner membrane into the periplasm, Nhead (60-325 aa) for adhesive function, FGG1 (326-363 aa) for seamless connection to the following domains, Cstalk (3219-3561 aa) as the C-terminal region of the PSD to ensure effective secretion, and TM (3562-3630 aa) to display the PSD on the surface of OM (Figure 3A). Note that PSD C-terminal regions are often involved in the secretion of the whole PSD through the TM in autotransporter families (Rojas-Lopez et al., 2017). The number of amino acids in mini-AtaA (775 aa) was reduced to 21% of that in FL-AtaA (3630 aa), and its molecular weight was only 76 kDa as a monomer. *E. coli* BL21 (DE3) cells were transformed with an expression plasmid for mini-AtaA or FL-AtaA, and their protein production was analyzed by SDS–PAGE, followed by immunoblotting using anti-AtaA_59-325_ antiserum. From the *E. coli* cells expressing the *FL-ataA* gene (*E. coli* (*FL-ataA*)), a faint band corresponding to the theoretical molecular weight of FL-AtaA was detected, but several bands corresponding to lower molecular weights were also detected, suggesting that most of the FL-AtaA was degraded (Figure 3B). From the *E. coli* cells expressing the *mini*-*ataA* gene (*E. coli* (*mini-ataA*)), a single band corresponding to the theoretical molecular weight of mini-AtaA was clearly detected. Subsequently, we examined their cell surface display by flow cytometry and immunofluorescence microscopy using anti-AtaA_59-325_ antiserum. The flow cytometry results clearly showed a much larger amount of Nhead on the cell surface of *E. coli* (*mini-ataA*) than on that of *E. coli* (*FL-ataA*) (Figure 3C). Immunofluorescence microscopy also showed that a higher amount of mini-AtaA was displayed on the *E. coli* cell surface compared to the weak expression signal of FL-AtaA (Figure 3D).

### Immobilization of *E. coli* cells onto a polyurethane foam support

**FIGURE 4:**
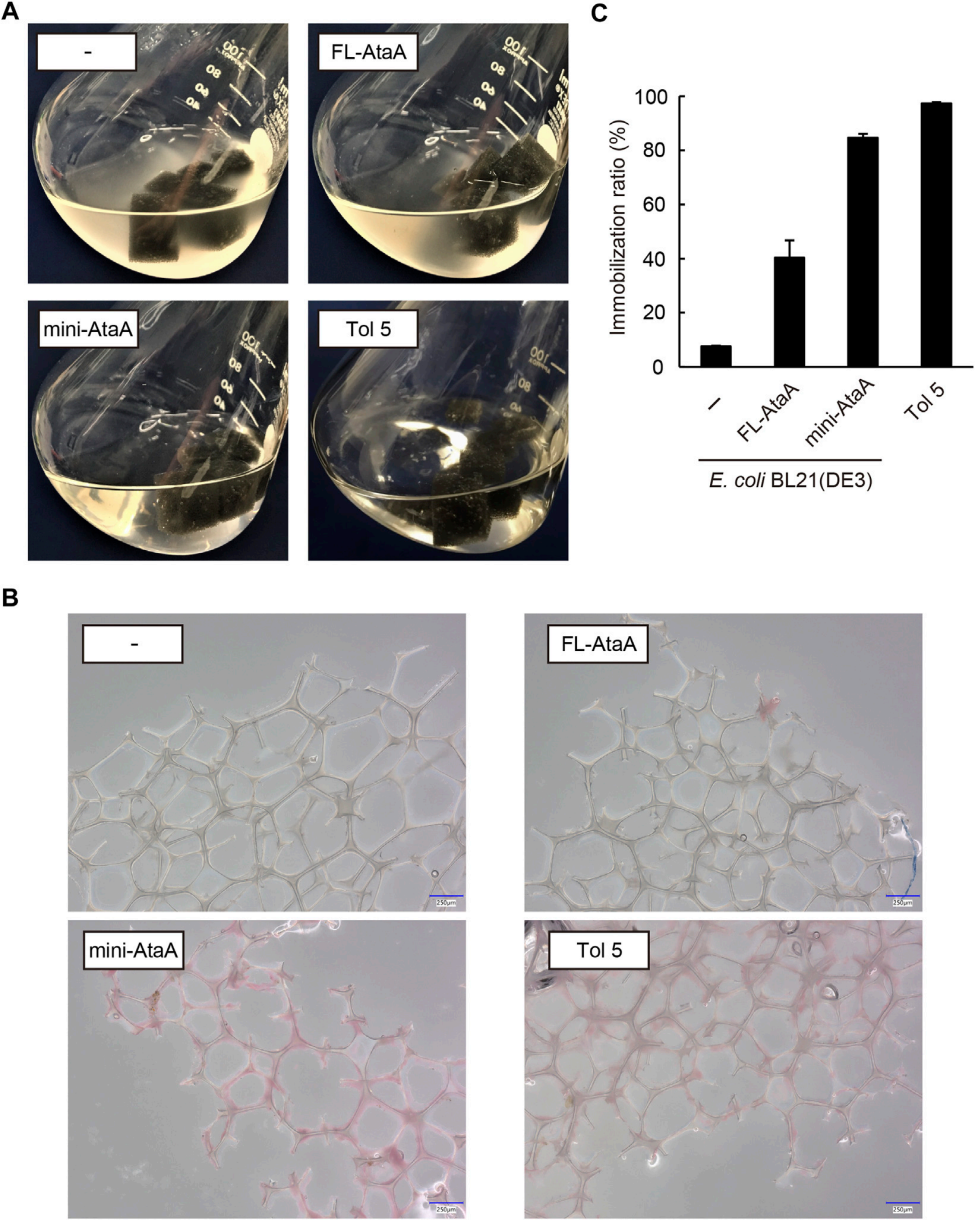
Immobilization of *E. coli* WT (−), *E. coli* (*FL-ataA*), and *E. coli* (*mini-ataA*) on the polyurethane foam support. **(A)** Photographs of the bacterial cell suspension after shaking for 60 min with the support. As a comparison, Tol 5, which originally expresses AtaA, is also shown. **(B)** Micrographs of polyurethane fibers after shaking with bacterial cells. The cells were stained with propidium iodide (red). Scale bars: 250 μm. **(C)** Immobilization ratio calculated from a decrease in the OD_600_ of the cell suspension after shaking. The data are represented as the mean ± SEM (n = 3).

We tried to immobilize the *E. coli* cells expressing mini-*ataA* onto a polyurethane foam, which is often used in bioprocesses as a support (Al-Amshawee et al., 2020). For comparison, *E. coli* wild type (*E. coli* WT), *E. coli* (*FL-ataA*)*,* and *Acinetobacter* sp. Tol 5 were also subjected to the cell immobilization experiment using polyurethane foam support (see materials and methods section). After shaking for 60 min in the presence of polyurethane foam support, *E. coli* WT and *E. coli* (*FL-ataA*) cell suspensions remained cloudy, indicating that many of the cells did not adhere to the polyurethane foam support (Figure 4A). In contrast, the cell suspension of *E. coli* (*mini-ataA*) became clear, as did Tol 5, suggesting that most of the cells adhered to the support. Microscopy revealed polyurethane fibers covered with adherent *E. coli* (*mini-ataA*) cells (Figure 4B). Then, the immobilization ratio was quantified based on the decrease in the OD_600_ of the cell suspension after shaking. While the immobilization ratios of *E. coli* WT and *E. coli* (*FL-ataA*) were only 8% and 40%, respectively, that of *E. coli* (*mini-ataA*) was 85% (Figure 4C). This immobilization ratio was slightly lower than the 97% of *Acinetobacter* sp. Tol 5 but significantly improved compared to that of *E. coli* (*FL-ataA*). Therefore, we achieved efficient immobilization of *E. coli* cells by the expression of a single recombinant gene, *mini-ataA*.

**FIGURE 5:**
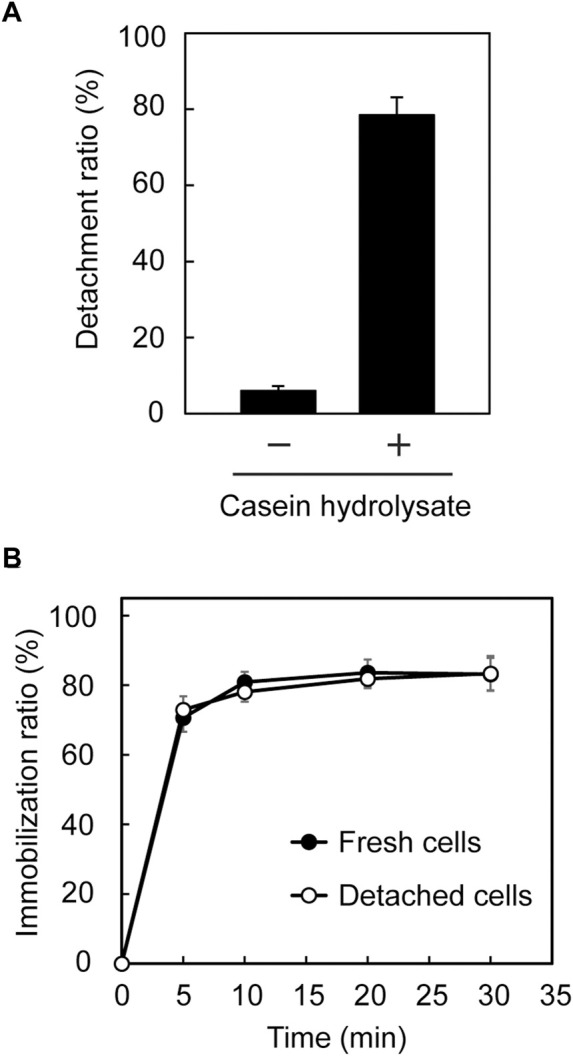
Detachment and re-immobilization of *E. coli* (*mini-ataA*) on the polyurethane foam support. **(A)** Detachment ratio calculated from an increase in the OD_600_ of the cell suspension after shaking in BS-N buffer with or without 1% casein hydrolysate. **(B)** Immobilization ratio calculated from a decrease in the OD_600_ of the cell suspension after shaking. The data are represented as the mean ± SEM (n = 3).

In our previous study (Ohara et al., 2019), immobilized Tol 5 cells on a polyurethane support were easily detached from the support by rinsing the cells with a solution containing casein hydrolysates, then the cells were re-immobilized onto the support without impairing their adhesiveness by removing the casein hydrolysates. Here, we examined whether a similar process is applicable to *E. coli* (*mini-ataA*). Five pieces of polyurethane foam support on which *E. coli* (*mini-ataA*) cells were immobilized were placed in 100-mL Erlenmeyer flasks containing 25 mL BS-N buffer supplemented with or without 1% casein hydrolysates. After 5 min of shaking, only 6% of *E. coli* (*mini-ataA*) cells detached from the support in BS-N buffer alone, but 78% of the cells detached in the BS-N buffer supplemented with 1% (w/v) casein hydrolysate (Figure 5A). Subsequently, the detached cells were collected by centrifugation, washed with BS-N buffer, resuspended in BS-N buffer at an OD_600_ of 1.0, and tested again for immobilization on the polyurethane foam support. The detached cells were immobilized as quickly as fresh cells, and the immobilization ratio reached 80% in 30 min (Figure 5B). These results imply that mini-AtaA retains adhesive properties similar to those of full-length AtaA, including a sensitivity to inhibitors of Nhead-surface interactions.

### Enzymatic reaction using *E. coli* cells expressing the *mini-ataA* gene

**FIGURE 6:**
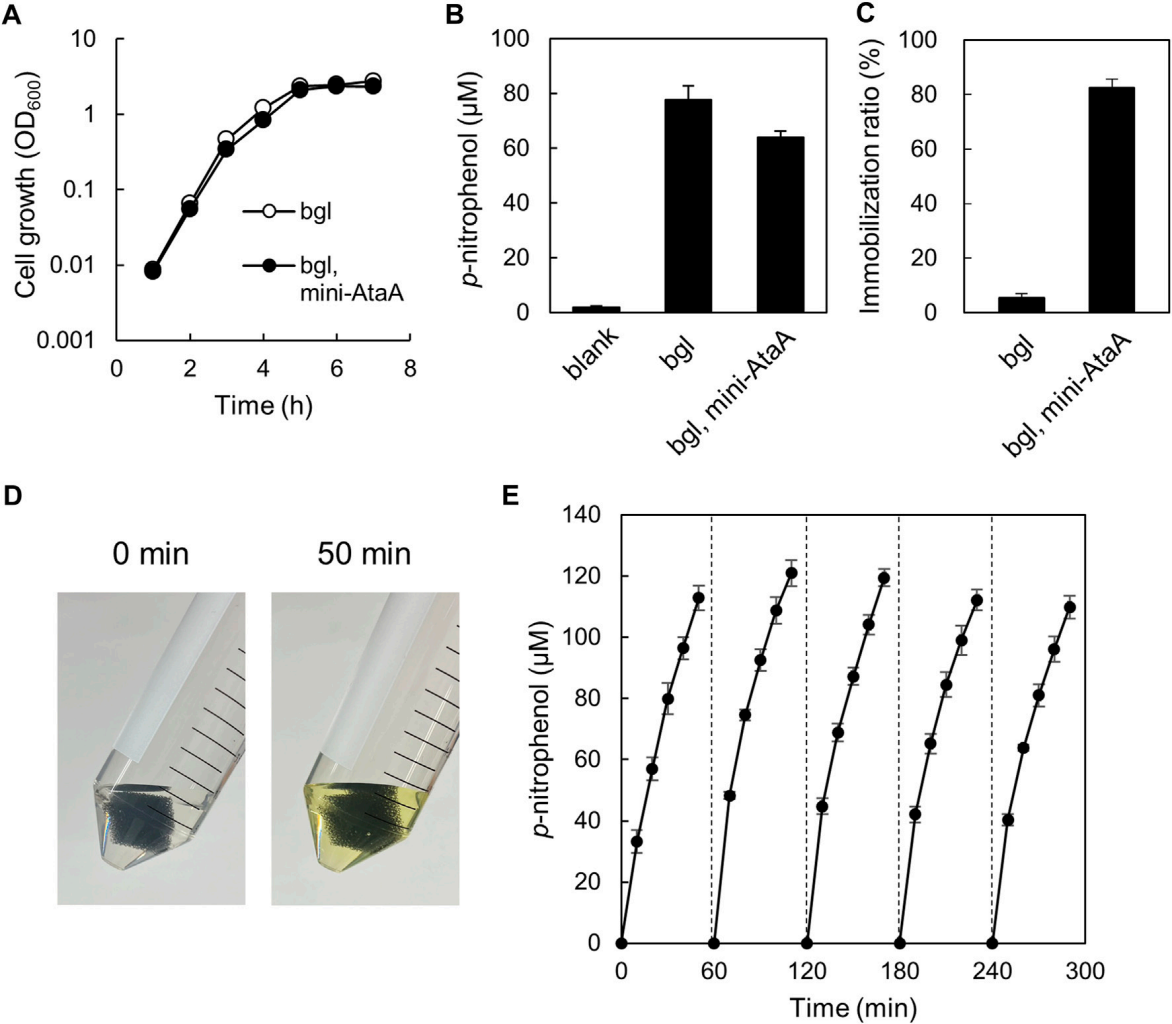
Enzymatic reaction by *E. coli* JCM (*bgl*, *mini-ataA*) cells expressing mini-AtaA and β-glucosidase. **(A)** Growth curves of *E. coli* JCM (*bgl*, *mini-ataA*) and the control strain harboring an empty vector (*bgl*) in LB medium supplemented with arabinose for induction of the *mini-ataA* gene. **(B)** BGL activity of suspended cells of *E. coli* JCM (*bgl*, *mini-ataA*) and of the control strain (*bgl*) harboring an empty vector. Blank, without cells. **(C)** Immobilization ratio calculated from a decrease in the OD_600_ of the cell suspension after shaking. **(D,E)** Repetitive BGL reaction using *E. coli* JCM (*bgl*, *mini-ataA*) cells. The support with the immobilized cells was placed in a test tube containing the reaction mixture. After every 50-min reaction, the support was collected, washed with BS-N buffer for 10 min, and transferred to a fresh reaction solution for the next new reaction. This process was repeated 5 times. Pictures of the test tubes before and after the 50-min reaction are shown in panel **(D)**. The data are represented as the mean ± SEM (n = 3).

We intended to enzymatic reaction by *E. coli* cells immobilized with mini-AtaA on polyurethane foam support. For this purpose, *E. coli* JCM-Tfu0937 (bgl)-Blc, the β-glucosidase gene-introduced *E. coli* strain JCM20137 (Tanaka et al., 2014), were transformed with the *mini-ataA* gene, generating *E. coli* JCM (*bgl*, *mini-ataA*). First, we examined the effect of *mini-ataA* expression on the growth of the *E. coli* cells and cellular BGL activity reflecting the production level of the enzyme. *E. coli* JCM (*bgl*, *mini-ataA*) showed a similar growth curve with the control cells harboring an empty vector (Figure 6A); the maximum specific growth rates (*μ*_max_) of JCM (*bgl*, *mini-ataA*) and the control cells were 1.9 h^−1^ and 2.0 h^−1^, respectively. The BGL activity of the cell suspensions was measured using 4-nitrophenyl *β*-*D*-glucopyranoside as a substrate. As a result, *E. coli* JCM (*bgl*, *mini-ataA*) showed approximately 80% BGL activity of the control cells (Figure 6B).

Next, JCM (*bgl*, *mini-ataA*) were immobilized on the polyurethane foam support, resulting in the similar immobilization ratio (>80%) (Figure 6C) as that of BL21 (DE3) expressing mini-AtaA (Figure 4C), although the control cells were hardly immobilized (Figure 6C). These immobilized cells were subjected to the repetitive enzymatic reaction to assess their reuse stability. One piece of the support with immobilized cells was incubated in BS-N buffer containing the substrate. After 50-min incubation at 37°C with shaking, the color of the reaction mixture was changed into yellow due to *p*-nitrophenol produced by BGL (Figure 6D). The absorbance at 405 nm was periodically measured during the incubation to obtain the *p*-nitrophenol concentration. Then, the support was picked up, washed with fresh BS-N buffer, transferred to a new reaction solution, and thereafter, another cycle of the reaction was repeated. The support with cells immobilized by expressing mini-AtaA maintained high BGL activity at least five cycles over a 5-h period (Figure 6E). These results demonstrate that the immobilized *E. coli* cells by mini-AtaA can be used for repetitive enzymatic reactions as a whole-cell catalyst.

## Discussion

In this study, the functional mapping of AtaA using IFD mutant genes revealed that the Nhead domain is responsible for the adhesive function and that other domains, including repeated sequences, are not essential for adhesion (Figure 2). Furthermore, mini-AtaA, which consists of Nhead, Cstalk and TM, exhibited adhesiveness (Figures 3, 4), while IFD-CPSD, which consists of Chead, Cstalk and TM, was reported to be non-adhesive in our previous study (Koiwai et al., 2016). These results clearly indicate that Nhead is the adhesive domain of AtaA. On the one hand, this is in agreement with findings for other TAAs, where the N-terminal head is usually involved in the adhesive function, e.g., for specific binding to the host extracellular matrix in the case of bacterial pathogens (Meuskens et al., 2019). On the other hand, the non-specific adhesion mechanisms of bacterial cell surface adhesins to abiotic surfaces are not fully understood, but it is generally assumed that non-specific adhesion is mediated by multipoint adsorption through repeated domains or combinations of domains with different adhesive properties (Frank et al., 2010; Boyd et al., 2014). In fact, some TAAs have been reported to bind to multiple types of biological substrates *via* different domains that specifically recognize each substrate (Heise and Dersch, 2006; Kaiser et al., 2012). Therefore, the non-specific, especially for abiotic surfaces, but extremely strong adhesion of AtaA (Ishii et al., 2022) that was achieved by the single domain Nhead is unique. It remains a challenge for future research to elucidate the molecular mechanism for the non-specific adhesion of Nhead.

Many secretory and chaperone proteins are involved in the secretion of outer membrane proteins, including TAAs, which are secreted by the type Vc secretion mechanism (Leo et al., 2012). In the type Vc secretion systems, a polypeptide chain is synthesized in the cytoplasm and passes through the inner membrane into the periplasm by the Sec system. The periplasmic chaperones DegP and Skp are involved in the periplasmic transit of polypeptides to prevent misfolding and aggregation (Grosskinsky et al., 2007; Ulrich et al., 2014). The TM of TAAs forms a 12-stranded β-barrel pore composed of three polypeptide chains (Meng et al., 2006; Shahid et al., 2012) and is inserted into the OM with the assistance of the β-barrel assembly machinery (Bam) complex (Lehr et al., 2010; Rooke et al., 2021). Finally, the PSD is transported to the cell surface through the β-barrel pore (Sikdar and Bernstein, 2019). These secretion systems are known to have some recognition specificity among species (Volokhina et al., 2013), and replacing the TM with a TM from related species can improve the efficiency of heterologous expression (Schmidgen et al., 2014). Furthermore, some highly specific periplasmic or lipoproteins, such as TpgA and SadB, are co-expressed with TAAs in some species to assist with their secretion (Grin et al., 2014; Ishikawa et al., 2016). When a TAA is heterologously expressed in a non-original host, the host secretion systems may not be able to recognize the polypeptide and will fail to secrete it, potentially resulting in the degradation of the misfolded polypeptide. Considering that the degradation of AtaA is reduced by deleting many parts of the PSD (Figure 3), one of the reasons for the increased cell-surface display of mini-AtaA may be due to the reduced chaperone dependence on secretion.

In this study, the disadvantages of the microbial cell immobilization method using the large AtaA protein have been avoided or mitigated by miniaturizing it on the basis of the functional mapping of AtaA domains; mini-AtaA expression is not a significant burden for *E. coli* cells. There was almost no effect of the production of mini-AtaA on the growth of *E. coli*. The mini-AtaA production reduced the activity of another enzyme, BGL, by 20%. However, this level of decrease in activity is acceptable in consideration of the high efficiency of bioprocesses achieved by cell immobilization, such as enabling repeated or continuous use of whole-cell catalysts and simplifying product separation. The drawback that the microorganisms to which this immobilization method can be applied are limited to Gram-negative bacteria is unavoidable due to the secretion mechanism of TAAs. Nevertheless, it is significant advance that the successful expression of *mini-ataA* in *E. coli* has achieved an immobilization efficiency comparable to that of the original *Acinetobacter* strain, Tol 5. *E. coli* is the most versatile platform for bioproduction because its metabolic pathways are well understood, many genetic engineering methods have been developed, and a variety of plasmids and host strains are commercially available. Thus, *E. coli* has been used for the bioproduction of a variety of chemicals, including amino acids, proteins, energy compounds, isoprenoids, and alkaloids (Huffer et al., 2012; Wang et al., 2017; Pontrelli et al., 2018; Gleizer et al., 2019; Yang et al., 2020; Rinaldi et al., 2022). Therefore, the immobilization of *E. coli* using *mini-ataA* will contribute to the expansion of environmentally friendly production processes using *E. coli* as whole-cell catalysts.

## Data Availability

The original contributions presented in the study are included in the article/Supplementary Material, further inquiries can be directed to the corresponding author.
