# Supplementary material for: Identification of the adhesive domain of AtaA from Acinetobacter sp. Tol 5 and its application in immobilizing Escherichia coli
Source: Front Bioeng Biotechnol. 2023 Jan 9;10:1095057. doi: 10.3389/fbioe.2022.1095057 (PMC9868564; doi:10.3389/fbioe.2022.1095057)
Supplement: Supplementary file 1 [file DataSheet1.PDF]

Supplementary Material

Table S1. Primers used in this study.

Name	Sequence (5'-3')
ΔNhead-f	CAGTTGGATAAAGCTTATGATGATAC
ΔNhead-r	AGCTGCTATTAAAGATAAAGGTGTTG
ΔNS-A1-f1	GATACCGCAGGTCTAAAATTTAAAG
ΔNS-A1-r1	TAAACGACCATTTGTATCATCATAAG
ΔNS-A2-f1	TGCAAGGATCCACAAACTATGAAGTTGCG
ΔNS-A2-r1	GGTCTCTACAGCATTTTTTGCATCTACAC
ΔNS-A2-f2	TTCGACGATTAGATCTGGTCTCGCTGTAAACACGTTAAATGCAGCG CAAG
ΔNS-A2-r2	AGTTCCGATCGTTGCTGCAGTACCGTTACCATTGC
ΔNS-B-f1	CGCGGATCCACAAACTATGAAGTTGCG
ΔNS-B-r1	CGCGGATCCAGCGACTACACTTCG
ΔNS-B-f2	GGTCTCGCGATCAACACGTTAAATGCAGCGCAAG
ΔNS-B-r2	GGATCCATCTGCATTGGTACC
ΔNS-CΔChead-f	TCACCGGTCTCCAATTAAAAGCGGTGGGTAACCA
ΔNS-CΔChead-r	TTGCAGGATCCAGCGACTACACTTCGTTGAGTTGC
ΔCstalk-f	AATACAACCAACCAAGCGGTAGTC
ΔCstalk-r	AACTTGGTTACCCACCGCTTTTAAC
mini-AtaA-f	ACTGCGCTTGATAACGCAATTAATAC
mini-AtaA-r	AATTGCATCATCAATCGTATTTTTACC

Table S2. Synthetic DNA fragments prepared in this study.

Name	Sequence (5'-3')
pTAKN-2::FragTrp3,4	GGTCTCGCTGTAGATACTGCAGGTCTAAAATTTAAAGGTGATACAGCAACCACAAGCAAT<br>ACCAAGAAATTAGGTGACACCGTTTCGATTACGGGTGATACGAACATTAGTACAGTTGCA<br>ACAACTGATGGTGTACAGGTTAAGTTAAATCCAAACTTAGATTTAGGAGCAACTGGTAGC<br>GTTAAAACGGGTAATACCACGATTAACAATGCAGGTGTAACAGCTGACCAAGTTACGGTT<br>GGTGGTGTTGTTATTAACAACACATCAGGTATTAATGCTGGTGGTAAAGCGATTACCAATG<br>TAGCAGCACCAACAAATAACACAGATGCTGCTAACAAGAAGTATGTAGATGACGCAGGT<br>ACAGCATTAACCAATTTGGGCTTTGGATTAAAAGCGCAAGATGGTACGACTGTGAACAA<br>GAAATTAGGTGAAGCAGTTGATATTGTTGGTTCAAACAGCAACATCAGTACAAAAGTAA<br>ATGCAGGCAAAGTAGAAGTTGCACTATCCAATACATTGGACTTAGGTACTACAGGTAGCG<br>TTACTACGGGTTCAACTGTAATTAACAATGCTGGTGTTACGGCAACTCAAGTTACCGCAA<br>ACAAAGTCACAGTTAATAATGCACCAACAGCAGGTACAGATGCGACCAATAAAACTTAT<br>GTAGACTCAAAAGCAGCGGCATCAAGAACAGAAGTCGCAGCTGGAAGCAATGTATCTG<br>GCGTAGTAAAAACGACAGGTGCAAACGGTCAAGACGTTTATACAGTAAATGCCAATGGT<br>ACGACTGCATCAGCAGGTTCTTCAGCAGTTACCGTAACACCAGGCACGAAAGATGCAAA<br>TAATGTCACTGACTATAAAGTAGACTTGTCAGCGACTACAAAAACCGATATTCAAAAAGG<br>TGTAGATGCAAAAAATGCTGTAGAGACC
pTAKN-2::FragTrp5,6	GGTCTCGCTGTAGATACCGCAGGTCTAAAATTTAAAGGTGATACAGCAACCACAAGCAAT<br>ACCAAGAAATTAGGTGACACCGTTTCGATTACGGGTGATACGAACATTAGTACAGTTGCG<br>ACAACTGATGGTGTACAGGTTAAGCTAAATCCAAACTTGGATTTAGGAGCAACTGGTAGC<br>GTTAAAACGGGTAATACCACGATTAACAATGCAGGTGTAACAGCTGATCAAGTTACAGTT<br>GGTGGTGTTGTTATTAACAACACATCAGGTATTAATGCTGGTGGTAAAGCGATTACCAATG<br>TAGCAGCACCAACAAATAACACAGATGCTGCTAACAAGAAGTATGTAGATGATGCAGGT<br>ACAGCATTAACCAATTTGGGCTTTGGATTAAAAGCGCAAGATGGTACGACTGTGAACAA<br>GAAATTAGGCGAAGCAGTTGAAGTTGTTGGTGCGGACAGTAACATCACCACGAAAGTTG<br>CAGGCGGTCAGGTTGCAATTGAGTTAAATAAAAACCTCAACAACTTAACTGGCATTACC<br>GTGAACGATGGAACCAATGGCACCAATGGATCC
pTAKN-2::Trp11_047&048	AGATCTACTGTGATTGGTAAAGATGGTATTTCGGTTAAAGATGGTTCAGGCAATACCATTG<br>CAGGTGTAGATAACACAGCGTTGACAGTTAAAGATGGCAGTGGCAACACAGAAACCAG<br>CATTAACCAAGCGATCAACACGTTAAATGCAGCGCAAGGTGAAACTGATAAGTTTGCAG<br>TGAAGTACGACAAAAATGCTGATGGCAGTGCAAACTATAACAATGTCACTTTAGCTGGTA<br>CAAATGGCACAATAATCAGCAATGTTAAAGCGGGTGCTGTGACCTCAACATCTACTGATG<br>CGATCAATGGTAGCCAATTA GAGACCGGATCC
pMD19::FragA	AGATCTAATATTGTCGTTACCCCGACGACAGCTTCTGATGGTTCAATATCGTATTCGGTTG<br>CTACAAGCGCAACACCGACGTTTACAAGTATAACTGTAAACAATGCACCAACGGCAGGT<br>ACAGATGCGACCAACAAGACTTATGTAGACTCAAAAGCAGCAGCATCGAGAACAGAAG<br>TAGCAGCTGGAAGCAATGTATCTGGTGTAGTAAAAACGACAGGCGCAAACGGTCAAGAC<br>GTTTATACAGTAAATGCCAATGGTACGACTGCATCAGCAGGTTCTTCAGCAGTTACCGTA<br>ACACCAGGCACGAAAGATGCAAATAATGTCACTGACTATAAAGTAGACTTATCAGCGACT<br>ACAAAAACCGATATCCAAAAAGGTGTAGATGCAAAAAATGCTGTAGATACCGCAGGTCT<br>AAAATTTAAAGGTGATACAGCAACCACAAGCAATACCAAGAAATTAGGTGACACCGTTT<br>CGATTACGGGTGATACGAACATTAGTACAGTTGCGACAACAGATGGTGTACAGGTTAAGT

	TAAATCCAAACTTGGATTTAGGAGCAACTGGTAGCGTTAAAACGGGTAATACCACGATTA<br>ACAATGCAGGTGTAACAGCTGATCAAGTTACGGTTGGTGGTGTTGTTATTAACAACACAT<br>CAGGTATTAATGCTGGTGGTAAAGCGATTACTAATGTAGCAGCACCAACAAATAACACAG<br>ATGCTGCTAACAAGAAGTATGTAGATGATGCAGGTACAGCATTAACCAATTTGGGCTTTG<br>GATTAAAAGCACAAGATGGTACGACTGTGAACAAGAAATTAGGTGAAGCAGTTGATATT<br>GTTGGTTCAAACAGCAACATCAGTACAAAAGTAAATGCAGGCAAAGTAGAAGTTGCACT<br>ATCCAATACATTGGACTTAGGTACTACAGGTAGCGTTACTACGGGTTCAACTGTAATTAAC<br>AATGCTGGTGTTACGGCAACTCAAGTTACCGCAAACAAAGTCACAATAAACAATGCACC<br>AACAGCAGGTACAGATGCGACCAACAAGACTTATGTAGACTCAAAAGCAGCAGCATCAA<br>GAACAGAAGTCGCAGCTGGAAGCAATGTATCTGGTGTAGTAAAAACGACAGGCGCAAA<br>CGGTCAAGATATTTATGCAGTAAATGCCAATGGTACGACTGCATCAGCAGGTTCTTCAGC<br>AGTTACCGTAACACCAGGCACGAAAGATGCAAATAATGTCACTGACTATAAAGTAGACTT<br>GTCAGCGACTACAAAAACCGATATTCAAAAAGGTGTAGATGCAAAAAATGCTGTAGATA<br>CTGCAGGTCTAAAATTTAAAGGTGATACAGCAACCACAAGCAATACCAAGAAATTAGGT<br>GACACCGTTTCGATTACGGGTGATACGAACATTAGTACAGTTGCAACAACTGATGGTGTA<br>CAGGTTAAGTTAAATCCAAACTTAGATTTAGGAGCAACTGGTAGCGTTAAAACGGGTAAT<br>ACCACGATTAACAATGCAGGTGTAACAGCTGACCAAGTTACGGTTGGTGGTGTTGTTATT<br>AACAACACATCAGGTATTAATGCTGGTGGTAAAGCGATTACCAATGTAGCAGCACCAACA<br>AATAACACAGATGCTGCTAACAAGAAGTATGTAGATGACGCAGGTACAGCATTAACCAAT<br>TTGGGCTTTGGATTAAAAGCGCAAGATGGTACGACTGTGAACAAGAAATTAGGTGAAGC<br>AGTTGATATTGTTGGTTCAAACAGCAACATCAGTACAAAAGTAAATGCAGGCAAAGTAG<br>AAGTTGCACTATCCAATACATTGGACTTAGGTACTACAGGTAGCGTTACTACGGGTTCAA<br>CTGTAATTAACAATGCTGGTGTTACGGCAACTCAAGTTACCGCAAACAAAGTCACAGTTA<br>ATAATGCACCAACAGCAGGTACAGATGCGACCAATAAAACTTATGTAGACTCAAAAGCA<br>GCGGCATCAAGAACAGAAGTCGCAGCTGGAAGCAATGTATCTGGCGTAGTAAAAACGAC<br>AGGTGCAAACGGTCAAGACGTTTATACAGTAAATGCCAATGGTACGACTGCATCAGCAG<br>GTTCTTCAGCAGTTACCGTAACACCAGGCACGAAAGATGCAAATAATGTCACTGACTATA<br>AAGTAGACTTGTCAGCGACTACAAAAACCGATATTCAAAAAGGTGTAGATGCAAAAAAT<br>GCTGTAGATACCGCAGGTCTAAAATTTAAAGGTGATACAGCAACCACAAGCAATACCAA<br>GAAATTAGGTGACACCGTTTCGATTACGGGTGATACGAACATTAGTACAGTTGCGACAAC<br>TGATGGTGTACAGGTTAAGCTAAATCCAAACTTGGATTTAGGAGCAACTGGTAGCGTTAA<br>AACGGGTAATACCACGATTAACAATGCAGGTGTAACAGCTGATCAAGTTACAGTTGGTGG<br>TGTTGTTATTAACAACACATCAGGTATTAATGCTGGTGGTAAAGCGATTACCAATGTAGCA<br>GCACCAACAAATAACACAGATGCTGCTAACAAGAAGTATGTAGATGATGCAGGTACAGC<br>ATTAACCAATTTGGGCTTTGGATTAAAAGCGCAAGATGGTACGACTGTGAACAAGAAATT<br>AGGCGAAGCAGTTGAAGTTGTTGGTGCGGACAGTAACATCACCACGAAAGTTGCAGGC<br>GGTCAGGTTGCAATTGAGTTAAATAAAAACCTCAACAACTTAACTGGCATTACCGTGAAC<br>GATGGAACCAATGGCACCAATGGATCC
pMD19::FragA_013	AGATCTAATATTGTCGTTACCCCGACGACAGCTTCTGATGGTTCAATATCGTATTCGGTTG<br>CTACAAGCGCAACACCGACGTTTACAAGTATAACTGTAAACAATGCACCAACGGCAGGT<br>ACAGATGCGACCAACAAGACTTATGTAGACTCAAAAGCAGCAGCATCGAGAACAGAAG<br>TAGCAGCTGGAAGCAATGTATCTGGTGTAGTAAAAACGACAGGCGCAAACGGTCAAGAC<br>GTTTATACAGTAAATGCCAATGGTACGACTGCATCAGCAGGTTCTTCAGCAGTTACCGTA<br>ACACCAGGCACGAAAGATGCAAATAATGTCACTGACTATAAAGTAGACTTATCAGCGACT<br>ACAAAAACCGATATCCAAAAAGGTGTAGATGCAAAAAATGCTGTAGATACCGCAGGTCT<br>AAAATTTAAAGGTGATACAGCAACCACAAGCAATACCAAGAAATTAGGTGACACCGTTT<br>CGATTACGGGTGATACGAACATTAGTACAGTTGCGACAACAGATGGTGTACAGGTTAAGT

	TAAATCCAAACTTGGATTTAGGAGCAACTGGTAGCGTTAAAACGGGTAATACCACGATTA
	ACAATGCAGGTGTAACAGCTGATCAAGTTACGGTTGGTGGTGTTGTTATTAACAACACAT
	CAGGTATTAATGCTGGTGGTAAAGCGATTACTAATGTAGCAGCACCAACAAATAACACAG
	ATGCTGCTAACAAGAAGTATGTAGATGATGCAGGTACAGCATTAACCAATTTGGGCTTTG
	GATTAAAAGCACAAGATGGTACGACTGTGAACAAGAAATTAGGTGAAGCAGTTGATATT
	GTTGGTTCAAACAGCAACATCAGTACAAAAGTAAATGCAGGCAAAGTAGAAGTTGCACT
	ATCCAATACATTGGACTTAGGTACTACAGGTAGCGTTACTACGGGTTCAACTGTAATTAAC
	AATGCTGGTGTTACGGCAACTCAAGTTACCGCAAACAAAGTCACAATAAACAATGCACC
	AACAGCAGGTACAGATGCGACCAACAAGACTTATGTAGACTCAAAAGCAGCAGCATCAA
	GAACAGAAGTCGCAGCTGGAAGCAATGTATCTGGTGTAGTAAAAACGACAGGCGCAAA
	CGGTCAAGATATTTATGCAGTAAATGCCAATGGTACGACTGCATCAGCAGGTTCTTCAGC
	AGTTACCGTAACACCAGGCACGAAAGATGCAAATAATGTCACTGACTATAAAGTAGACTT
	GTCAGCGACTACAAAAACCGATATTCAAAAAGGTGTAGATGCAAAAAATGCTGTAGATA
	CTGCAGGTCTAAAATTTAAAGGTGATACAGCAACCACAAGCAATACCAAGAAATTAGGT
	GACACCGTTTCGATTACGGGTGATACGAACATTAGTACAGTTGCAACAACTGATGGTGTA
	CAGGTTAAGTTAAATCCAAACTTAGATTTAGGAGCAACTGGTAGCGTTAAAACGGGTAAT
	ACCACGATTAACAATGCAGGTGTAACAGCTGACCAAGTTACGGTTGGTGGTGTTGTTATT
	AACAACACATCAGGTATTAATGCTGGTGGTAAAGCGATTACCAATGTAGCAGCACCAACA
	AATAACACAGATGCTGCTAACAAGAAGTATGTAGATGACGCAGGTACAGCATTAACCAAT
	TTGGGCTTTGGATTAAAAGCGCAAGATGGTACGACTGTGAACAAGAAATTAGGTGAAGC
	AGTTGATATTGTTGGTTCAAACAGCAACATCAGTACAAAAGTAAATGCAGGCAAAGTAG
	AAGTTGCACTATCCAATACATTGGACTTAGGTACTACAGGTAGCGTTACTACGGGTTCAA
	CTGTAATTAACAATGCTGGTGTTACGGCAACTCAAGTTACCGCAAACAAAGTCACAGTTA
	ATAATGCACCAACAGCAGGTACAGATGCGACCAATAAAACTTATGTAGACTCAAAAGCA
	GCGGCATCAAGAACAGAAGTCGCAGCTGGAAGCAATGTATCTGGCGTAGTAAAAACGAC
	AGGTGCAAACGGTCAAGACGTTTATACAGTAAATGCCAATGGTACGACTGCATCAGCAG
	GTTCTTCAGCAGTTACCGTAACACCAGGCACGAAAGATGCAAATAATGTCACTGACTATA
	AAGTAGACTTGTCAGCGACTACAAAAACCGATATTCAAAAAGGTGTAGATGCAAAAAAT
	GCTGTAGATACCGCAGGTCTAAAATTTAAAGGTGATACAGCAACCACAAGCAATACCAA
	GAAATTAGGTGACACCGTTTCGATTACGGGTGATACGAACATTAGTACAGTTGCGACAAC
	TGATGGTGTACAGGTTAAGCTAAATCCAAACTTGGATTTAGGAGCAACTGGTAGCGTTAA
	AACGGGTAATACCACGATTAACAATGCAGGTGTAACAGCTGATCAAGTTACAGTTGGTGG
	TGTTGTTATTAACAACACATCAGGTATTAATGCTGGTGGTAAAGCGATTACCAATGTAGCA
	GCACCAACAAATAACACAGATGCTGCTAACAAGAAGTATGTAGATGATGCAGGTACAGC
	ATTAACCAATTTGGGCTTTGGATTAAAAGCGCAAGATGGTACGACTGTGAACAAGAAATT
	AGGCGAAGCAGTTGAAGTTGTTGGTGCGGACAGTAACATCACCACGAAAGTTGCAGGC
	GGTCAGGTTGCAATTGAGTTAAATAAAAACCTCAACAACTTAACTGGCATTACCGTGAAC
	GATGGAACCAATGGCACCAATGGATCTACTGTGATTGGTAAAGATGGTATTTCGGTTAAA
	GATGGTTCAGGCAATACCATTGCAGGTGTAGATAACACAGCGTTGACAGTTAAAGATGGC
	AGTGGCAACACAGAAACCAGCATTAACCAAGCGATCGAGACCGGATCC
pMD19::FragB	GAAACAGCTATGACCATGATTACGCCAAGTTTGCACGCCTGCCGTTCGACGATTAGATCT
	ACTGTGATTGGTAAAGATGGTATTTCGGTTAAAGATGGTTCAGGCAATACCATTGCAGGT
	GTAGATAACACAGCGTTGACAGTTAAAGATGGCAGTGGCAACACAGAAACCAGCATTAA
	CCAAGCGATCAACACGTTAAATGCAGCGCAAGGTGAAACTGATAAGTTTGCAGTGAAGT
	ACGACAAAAATGCTGATGGCAGTGTGAACTACAACAACATCACATTGGCAGGTACGACT
	GCAAGCAGTACACAAGATGCAACTACAGGCAAGATCACCACAACAGGTGGAACAAGCT

TGAACAATGTTGCAAGTGCGGGTGACTACAAAGATGTTGCCAATGCAAGCAAAGGTGTA
AACGCAGGTGACTTAAACAATGCAGTTGTTGATGCAACCAATGCAGCAACCAGCAAAGG
CTTTGCATTACAAGCAGCAGATGGCGCTAAAGTTCAGAAGAACCTAGGCGAAGCAGTTG
AAGTTGTCGGTGCCGACAGCAACATCACCACAAAAGTTGCAGGCGGTCAGGTTGCAATT
GAGTTAAATAAAAACCTCAACAACTTAACTGGCATTACCGTGAACGATGGAACCAATGG
CACCAATGGTTCAACTGTGATTGGTAAAGATGGTATTTCAGTTAAAGACGGTTCAGGCAA
TACCATTGCAGGTGTAGATAACACAGCGTTGACAGTTAAAGATGGCAGTGGCAACACAG
AAACCAGCATTAACCAAGCGATCAACACGTTAAATGCAGCGCAAGGTGAAACTGATAAG
TTTGCAGTGAAGTACGACAAAAATACGGATGGTAGTACCAACTACAACAGTATTACTGCA
GGCAATGGTAACGGTACTGCAGCAACGATCGGAACTGACACAGCAGGTAATAGTGTTGT
GACCAGTGGCGGAACTAAAATTAGTAATGTTGCGAATGGTGTCAATGCAAGTGATGCAG
TAAACAAAGGTCAATTGGATAGCTTAAGTACAGGTCTTACCAATACAGGCTTTGGTTTAA
AAGCAGCAGATGGCAACACCGTTAACAAAAAATTAGGCGAAGCAGTAGACGTTGTCGG
TGCTGACAGCAACATCACCACGAAAGTTGCAGGCGGTCAGGTTGCGATTGAGTTAAATA
AAAACCTCAACAACTTAACTGGCATTACCGTGAACGATGGAACCAATGGCACCAATGGT
TCAACTGTGATTGGTAAAGATGGTATTTCGATTAAAGATGGTTCAGGCAATACCATTGCAG
GTGTAGATAACACAGCGTTGACAGTTAAAGATGGCAGTGGCAACACAGAAACCAGCATT
AACCAAGCGATCAACACGTTAAATGCAGCGCAAGGTGAAACTGACAAGTTTGCAGTGA
AGTACGACAAGAATGCTGATGGCAGTGCAAACTACAACAACATCACATTGGCAGGTACG
ACTGCAAGTAGCACGCAAGATGCAACAACAGGCAAGATCACCACAACAGGTGGAACAA
GCTTGAACAACGTTGCAAGTGCAGGTGACTACAAAGATGTTGCCAATGCAAGCAAAGGT
GTAAACGCAGGTGACTTGAACAATGCAGTTGTTGATGCAACCAATGCAGCAACCAGCAA
AGGCTTTGCATTACAAGCAGCAGATGGCGCTAAAGTTCAGAAGAACCTAGGCGAAGCAG
TTGAAGTTGTCGGTGCGGACAGCAACATCACCACAAAAGTAGTGGGTGGACAAGTTGC
GATTGAGTTAAATAAAAACCTCAACAACTTAACTGGCATTACCGTGAACGATGGAACCA
ATGGCACAAATGGTTCAACTGTGATTGGTAAAGATGGTATTTCGGTTAAAGATGGTTCAG
GTAATACCATTGCAGGTGTAGATAACACAGCGTTGACAGTTAAAGATGGCAGTGGCAAC
ACAGAAACCAGCATTAACCAAGCGATCAACACGTTAAATGCAGCGCAAGGTGAAACTG
ATAAGTTTGCAGTGAAGTACGACAAAAATGCTGATGGCAGTGTGAACTACAACAACATC
ACATTGGCAGGTACGACTGCAAGCAGTACACAAGATGCAACTACAGGCAAGATCACCAC
AACAGGTGGAACAAGCTTGAACAATGTTGCAAGTGCGGGTGACTACAAAGATGTTGCC
AATGCAAGCAAAGGTGTAAACGCAGGTGACTTAAACAATGCAGTTGTTGATGCAACCAA
TGCAGCAACCAGCAAAGGCTTTGCATTACAAGCAGCAGATGGCGCTAAAGTTCAGAAGA
ACCTAGGCGAAGCAGTTGAAGTTGTCGGTGCCGACAGCAACATCACCACAAAAGTTGC
AGGCGGTCAGGTTGCAATTGAGTTAAATAAAAACCTCAACAACTTAACTGGCATTACCGT
GAACGATGGAACCAATGGCACCAATGGTTCAACTGTGATTGGTAAAGATGGTATTTCAGT
TAAAGACGGTTCAGGCAATACCATTGCAGGTGTAGATAACACAGCGTTGACAGTTAAAG
ATGGCAGTGGCAACACAGAAACCAGCATTAACCAAGCGATCAACACGTTAAATGCAGCG
CAAGGTGAAACTGATAAGTTTGCAGTGAAGTACGACAAAAATGCTGATGGCAGTGTGAA
CTACAACAACATCACATTGGCAGGTACGACTGCAAGCAGTACACAAGATGCAACTACAG
GCAAGATCACCACAACAGGTGGTACAAGCTTGAACAATGTTGCAAGTGCGGGTGACTAC
AAAGATGTTGCCAATGCAAGCAAAGGTGTAAACGCAGGTGACTTGAACAATGCAGTTGT
TGATGCAACCAATGCAGCGACCAGCAAAGGCTTTGCATTACAAGCAGCAGATGGCGCTA
AAGTTCAGAAGAACCTAGGCGAAGCAGTTGAAGTTGTTGGTGCGGACAGTAACATCACC
ACGAAAGTTGCAGGCGGTCAGGTTGCAATTGAGTTAAATAAAAACCTCAACAACTTAAC
TGGCATTACCGTGAACGATGGAACCAATGGCACCAATGGATCCAATCTCTGGAAGATCCG
CGCGTACCGAGTTCTAATTCACTGGCCGTCGTTTTACAACGTCGTGACTGGGAAAACCCT
GGCGTTACCC

pMD19::FragC	CAGGAAACAGCTATGACCATGATTACGCCAAGTTTGCACGCCTGCCGTTCGACGATTAGA<br>TCTACTGTGATTGGTAAAGATGGTATTTCGGTTAAAGATGGTTCAGGCAATACCATTGCAG<br>GTGTAGATAACACAGCGTTGACAGTTAAAGATGGCAGTGGCAACACAGAAACCAGCATT<br>AACCAAGCGATCAACACGTTAAATGCAGCGCAAGGTGAAACTGATAAGTTTGCAGTGAA<br>GTACGACAAAAATGCTGATGGCAGTGCAAACTATAACAATGTCACTTTAGCTGGTACAAA<br>TGGCACAATAATCAGCAATGTTAAAGCGGGTGCTGTGACCTCAACATCTACTGATGCGAT<br>CAATGGTAGCCAATTATATGGTGTTGCAAACAGCGTGAAGAATGCAATTGGTGGTTCAAC<br>CACAATTGATGCAACGACTGGTGCAATCACGACGACCAATATTGGTGGTACAGGTTCAA<br>ATACGATTGATGGTGCAATCAGCAGTATTAAAGATTCAGCGACTAAAGCGAAAACCACG<br>GTAAGTGCTGGGGATAATGTTGTCGTTACATCGGGTACCAATGCAGATGGCTCAACAAAC<br>TATGAAGTTGCGACAGCGAAAGACGTTAACTTTGACAAAGTGACTGTAGGTAGTGTTGT<br>TGTAGATAAATCAAGCAATACAATCAAAGGATTAAGTAATACCACTTGGAACGGAACAGC<br>AGTATCAGGTCAAGCGGCGACAGAAGACCAGTTAAAAACGGTCAGCGATGCGCAAGGT<br>GAAACTGATAAGTTTGCAGTGAAGTACGACAAAAATGCTGATGGCAGTGCGAACTACAA<br>CAGTATTACTGCAGGCAATGGTAACGGTACTGCAGCAACGATCGGAACTGACACAGCAG<br>GTAATAGTGTTGTGACCAGTGGCGGAACTAAAATTAGTAATGTTGCGAATGGTGTCAATG<br>CAAGTGATGCAGTAAACAAAGGTCAATTGGATAGCTTAAGTACAGGTCTTACCAATACAG<br>GCTTTGGTTTAAAAGCAGCAGATGGCAACACCGTTAACAAAAAATTAGGCGAAGCAGTA<br>GACGTTGTCGGTGCTGACAGCAACATCACCACGAAAGTTGCAGGCGGTCAGGTTGCGAT<br>TGAGTTAAATAAAAACCTCAACAACTTAACTGGCATTACCGTGAACGATGGAACCAATG<br>GCACCAATGGTTCAACTGTGATTGGTAAAGATGGTATTTCGATTAAAGATGGTTCAGGCA<br>ATACCATTGCAGGTGTAGATAACACAGCGTTGACGGTTAAAGATAGCAGTGGCAACACA<br>GAAACCAGCATTAACCAAGCGATCAACACGTTAAATGCAGCGCAAGGTGAAACTGATAA<br>GTTTGCAGTGAAGTACGATAAGAATGCTGATGGCAGTGTGAACTATAACAATGTCACTTT<br>AGCAGGTACAAATGGCACAATAATCAGAAATGTTAAAGCGGGTGCTGTGACCTCAACAT<br>CTACTGATGCGATCAATGGTAGCCAATTATACGATATTGCAAACAGCGTGAAGAATGCAAT<br>TGGTGGTTCAACCACAAGAGATGTAACGACTGGTGCAATCACAACGACCAATATTGGTG<br>GTACAGGTTCAAACACGATTGATGGTGCAATCAGCAGTATTAAAGATTCAGCGACTAAAG<br>CGAAAACCACGATAAGTGCTGGGGATAATGTTGTCGTTACATCGGGTACCAATGCAGATG<br>GATCCAATCTCTGGAAGATCCGCGCGTACCGAGTTCTAATTCACTGGCCGTCGTTTTACA<br>ACGTCGTGAC
pMD19::FragA_013	AGATCTAATATTGTCGTTACCCCGACGACAGCTTCTGATGGTTCAATATCGTATTCGGTTG<br>CTACAAGCGCAACACCGACGTTTACAAGTATAACTGTAAACAATGCACCAACGGCAGGT<br>ACAGATGCGACCAACAAGACTTATGTAGACTCAAAAGCAGCAGCATCGAGAACAGAAG<br>TAGCAGCTGGAAGCAATGTATCTGGTGTAGTAAAAACGACAGGCGCAAACGGTCAAGAC<br>GTTTATACAGTAAATGCCAATGGTACGACTGCATCAGCAGGTTCTTCAGCAGTTACCGTA<br>ACACCAGGCACGAAAGATGCAAATAATGTCACTGACTATAAAGTAGACTTATCAGCGACT<br>ACAAAAACCGATATCCAAAAAGGTGTAGATGCAAAAAATGCTGTAGATACCGCAGGTCT<br>AAAATTTAAAGGTGATACAGCAACCACAAGCAATACCAAGAAATTAGGTGACACCGTTT<br>CGATTACGGGTGATACGAACATTAGTACAGTTGCGACAACAGATGGTGTACAGGTTAAGT<br>TAAATCCAAACTTGGATTTAGGAGCAACTGGTAGCGTTAAAACGGGTAATACCACGATTA<br>ACAATGCAGGTGTAACAGCTGATCAAGTTACGGTTGGTGGTGTTGTTATTAACAACACAT<br>CAGGTATTAATGCTGGTGGTAAAGCGATTACTAATGTAGCAGCACCAACAAATAACACAG<br>ATGCTGCTAACAAGAAGTATGTAGATGATGCAGGTACAGCATTAACCAATTTGGGCTTTG<br>GATTAAAAGCACAAGATGGTACGACTGTGAACAAGAAATTAGGTGAAGCAGTTGATATT

	GTTGGTTCAAACAGCAACATCAGTACAAAAGTAAATGCAGGCAAAGTAGAAGTTGCACT<br>ATCCAATACATTGGACTTAGGTACTACAGGTAGCGTTACTACGGGTTCAACTGTAATTAAC<br>AATGCTGGTGTTACGGCAACTCAAGTTACCGCAAACAAAGTCACAATAAACAATGCACC<br>AACAGCAGGTACAGATGCGACCAACAAGACTTATGTAGACTCAAAAGCAGCAGCATCAA<br>GAACAGAAGTCGCAGCTGGAAGCAATGTATCTGGTGTAGTAAAAACGACAGGCGCAAA<br>CGGTCAAGATATTTATGCAGTAAATGCCAATGGTACGACTGCATCAGCAGGTTCTTCAGC<br>AGTTACCGTAACACCAGGCACGAAAGATGCAAATAATGTCACTGACTATAAAGTAGACTT<br>GTCAGCGACTACAAAAACCGATATTCAAAAAGGTGTAGATGCAAAAAATGCTGTAGATA<br>CTGCAGGTCTAAAATTTAAAGGTGATACAGCAACCACAAGCAATACCAAGAAATTAGGT<br>GACACCGTTTCGATTACGGGTGATACGAACATTAGTACAGTTGCAACAACTGATGGTGTA<br>CAGGTTAAGTTAAATCCAAACTTAGATTTAGGAGCAACTGGTAGCGTTAAAACGGGTAAT<br>ACCACGATTAACAATGCAGGTGTAACAGCTGACCAAGTTACGGTTGGTGGTGTTGTTATT<br>AACAACACATCAGGTATTAATGCTGGTGGTAAAGCGATTACCAATGTAGCAGCACCAACA<br>AATAACACAGATGCTGCTAACAAGAAGTATGTAGATGACGCAGGTACAGCATTAACCAAT<br>TTGGGCTTTGGATTAAAAGCGCAAGATGGTACGACTGTGAACAAGAAATTAGGTGAAGC<br>AGTTGATATTGTTGGTTCAAACAGCAACATCAGTACAAAAGTAAATGCAGGCAAAGTAG<br>AAGTTGCACTATCCAATACATTGGACTTAGGTACTACAGGTAGCGTTACTACGGGTTCAA<br>CTGTAATTAACAATGCTGGTGTTACGGCAACTCAAGTTACCGCAAACAAAGTCACAGTTA<br>ATAATGCACCAACAGCAGGTACAGATGCGACCAATAAAACTTATGTAGACTCAAAAGCA<br>GCGGCATCAAGAACAGAAGTCGCAGCTGGAAGCAATGTATCTGGCGTAGTAAAAACGAC<br>AGGTGCAAACGGTCAAGACGTTTATACAGTAAATGCCAATGGTACGACTGCATCAGCAG<br>GTTCTTCAGCAGTTACCGTAACACCAGGCACGAAAGATGCAAATAATGTCACTGACTATA<br>AAGTAGACTTGTCAGCGACTACAAAAACCGATATTCAAAAAGGTGTAGATGCAAAAAAT<br>GCTGTAGATACCGCAGGTCTAAAATTTAAAGGTGATACAGCAACCACAAGCAATACCAA<br>GAAATTAGGTGACACCGTTTCGATTACGGGTGATACGAACATTAGTACAGTTGCGACAAC<br>TGATGGTGTACAGGTTAAGCTAAATCCAAACTTGGATTTAGGAGCAACTGGTAGCGTTAA<br>AACGGGTAATACCACGATTAACAATGCAGGTGTAACAGCTGATCAAGTTACAGTTGGTGG<br>TGTTGTTATTAACAACACATCAGGTATTAATGCTGGTGGTAAAGCGATTACCAATGTAGCA<br>GCACCAACAAATAACACAGATGCTGCTAACAAGAAGTATGTAGATGATGCAGGTACAGC<br>ATTAACCAATTTGGGCTTTGGATTAAAAGCGCAAGATGGTACGACTGTGAACAAGAAATT<br>AGGCGAAGCAGTTGAAGTTGTTGGTGCGGACAGTAACATCACCACGAAAGTTGCAGGC<br>GGTCAGGTTGCAATTGAGTTAAATAAAAACCTCAACAACTTAACTGGCATTACCGTGAAC<br>GATGGAACCAATGGCACCAATGGATCTACTGTGATTGGTAAAGATGGTATTTCGGTTAAA<br>GATGGTTCAGGCAATACCATTGCAGGTGTAGATAACACAGCGTTGACAGTTAAAGATGGC<br>AGTGGCAACACAGAAACCAGCATTAACCAAGCGATCGAGACCGGATCC
pDONR221::FragA2_031	ATGAATAAAATCTACAAAGTGATTTGGAATGCGACTTTGTTGGCATGGGTTGCAGTATCT<br>GAATTGGCAAAAGGGAAAACCAAATCTACGACATCAAAATCCAAAGCTAAATCATTATCT<br>TCATCTGTAATAGTTGGTGGGATAATATTAACAACACCTTTATCTTTAATAGCAGCTACTGT<br>TCAAGTTGGAGGGGGAACTAATTCTGGAACAACTGCTACAGCTTCTACGAATTGTGCAG<br>ACTTATATAATTATCAAAATCCTGAGAACTCAGGCTCTGGAGCGGCTGGGAATTATAATGC<br>AGGAAATCCAAGTGTGTGTTCGATCGCTATAGGTGAAAACGCACAAGGTGGTACTTCTG<br>GAACTGGAGGGTCGCCAGGGATAGCGATAGGTGGAAATTCTAAAGCTACGGGTGGTTTA<br>TCTGTTGCTATAGGCGGATATGCTCAAGCGACAAATGTTGGAAGTATTGCTTTAGGCACA<br>GCAGCTTTATCAAGTGGTTTTAACAGTTTAGCAATATCCAGACAAGCTGCTGCAACGAAT<br>AACTATTCAATAGCTATAGGTACAACTTCAGTTTCGAAAGGAGTTGGATCGATTGCTATGG<br>GGCATTCAACGAATGCTTCTGGAGATCAATCGATAGCAATTGGTAGCTCGGATGCTGTTA<br>ATTCAGCAACAGCAACAACAACATACGATGGTACAACAAATACTCAAGCATCAGGTAGT

AAATCGATTGCTATAGGTGCAAGCGCAAAGGCATCAACCAATAACAGCATTGCACTAGGT
GCAGGATCGGTAACTTCTGCACAATCTGGTAATTCTTATCTTACTGGTGTAGGTGCATCAG
CTACAAATGGTGTTGTATCTGTTGGAACTTCAACTGCAACACGTCGTATCCAAAATGTAG
CAGATGGTTCAGCCGCTTCAGATGCTGTGACAGTTGCTCAGTTGGATAAAGCTTATGATG
ATACAAATGGTCGTTTAGCTGCTGCTTTAGGTACAGGTAGTGGTGCTGCCTATAATGCAGC
AAACAATACATATACCGCTCCAACGAATATTGGGGGAACAGGTAAAAATACGATTGATGA
TGCAATTAAAGCAACTCAACGAAGTGTAGTCGCTGGATCTAATATTGTCGTTACCCCGAC
GACAGCTTCTGATGGTTCAATATCGTATTCGGTTGCTACAAGCGCAACACCGACGTTTAC
AAGTATAACTGTAAACAATGCACCAACGGCAGGTACAGATGCGACCAACAAGACTTATG
TAGACTCAAAAGCAGCAGCATCGAGAACAGAAGTAGCAGCTGGAAGCAATGTATCTGGT
GTAGTAAAAACGACAGGCGCAAACGGTCAAGACGTTTATACAGTAAATGCCAATGGTAC
GACTGCATCAGCAGGTTCTTCAGCAGTTACCGTAACACCAGGCACGAAAGATGCAAATA
ATGTCACTGACTATAAAGTAGACTTATCAGCGACTACAAAAACCGATATCCAAAAAGGTG
TAGATGCAAAAAATGCTGTAGATACCGCAGGTCTAAAATTTAAAGGTGATACAGCAACCA
CAAGCAATACCAAGAAATTAGGTGACACCGTTTCGATTACGGGTGATACGAACATTAGTA
CAGTTGCGACAACAGATGGTGTACAGGTTAAGTTAAATCCAAACTTGGATTTAGGAGCA
ACTGGTAGCGTTAAAACGGGTAATACCACGATTAACAATGCAGGTGTAACAGCTGATCAA
GTTACGGTTGGTGGTGTTGTTATTAACAACACATCAGGTATTAATGCTGGTGGTAAAGCG
ATTACTAATGTAGCAGCACCAACAAATAACACAGATGCTGCTAACAAGAAGTATGTAGAT
GATGCAGGTACAGCATTAACCAATTTGGGCTTTGGATTAAAAGCACAAGATGGTACGACT
GTGAACAAGAAATTAGGTGAAGCAGTTGATATTGTTGGTTCAAACAGCAACATCAGTAC
AAAAGTAAATGCAGGCAAAGTAGAAGTTGCACTATCCAATACATTGGACTTAGGTACTAC
AGGTAGCGTTACTACGGGATCTACTGTAATTAACAATGCTGGTGTTACGGCAACTCAAGT
TACCGCAAACAAAGTCACAATAAACAATGCACCAACAGCAGGTACAGATGCGACCAAC
AAGACTTATGTAGACTCAAAAGCAGCAGCATCAAGAACAGAAGTCGCAGCTGGAAGCA
ATGTATCTGGTGTAGTAAAAACGACAGGCGCAAACGGTCAAGATATTTATGCAGTAAATG
CCAATGGTACGACTGCATCAGCAGGTTCTTCAGCAGTTACCGTAACACCAGGCACGAAA
GATGCAAATAATGTCACTGACTATAAAGTAGACTTGTCAGCGACTACAAAAACCGATATT
CAAAAAGGTGTAGATGCAAAAAATGCTGTAGAGACCGGATCCACAAACTATGAAGTTGC
GACAGCGAAAGACGTTAACTTTGACAAAGTAACTGTAGGTAATGTTGTTGTTGATAAGG
CAAATGACACGATCCAAGGTTTGAGCAATAAAGATCTAAATTCAACTGATTTTGCGACCA
AAGGTAGAGCTGCGACTGAAGAACAGTTAAAAGCAGTGATTACCAGTAATATCACGGAA
GTTGTGGATGGTAATGGCAACAAGGTGAATATTATTGACCAAGTTGTAAATACCAAACCT
GACAATAAGAACCAAGATTCATTGTTCTTAACGTATGACAAACAAGGTCAAGAAACCAC
AGATCGCCTAACGATTGGTCAAACGGTACAGAAGATGAATACTGATGGTATTAAATTCTT
CCATACCAATGCCGATACATCAAAAGGTGATTTGGGTACAACAAATGACTCAAGTGCAGG
TGGTTTAAACTCTACAGCAATTGGTGTAAATGCGATTGTTGCGAATGGTGCAGATAGTTC
AGTTGCTTTAGGTCATAACACCAAAGTCAATGGTAAACAATCAATTGCAATTGGTTCTGG
TGCAGAAGCTTTAGGCAATCAATCGATCAGTATTGGTACAGGCAATAAAGTCACTGGTGA
TCATTCGGGTGCGATTGGTGATCCAACTATTGTAAATGGTGCAAACAGCTACTCTGTGGG
TAATAACAACCAAGTACTTACAGATGACACTTTCGTACTTGGAAACAATGTCACCAAAAC
TATTGCTGGTTCAGTAGTATTGGGTAACGGTTCAGCTGCAACGACAGGTGCTGGTGAGGC
AGGCTATGCCTTATCTGTAGCAACAAATGCAGATAAAGCCGCGATCACTAAAACTACGTC
AAGCACTGGTGCTGTTGCAGTTGGTGATGCGTCGAGCGGTATTTATCGTCAAATTACCGG
TGTTGCTGCGGGTAGCGTAGATTCAGATGCTGTGAACGTTGCACAGTTAAAAGCGGTGG
GTAACCAAGTTGTAACGACTCAAACTACATTGGTGAACAGTTTGGGTGGTAACGCTAAA
GTAAATGCAGACGGTACGATTACAGGACCAACTTATAATGTTGCTCAAGGTAATCAGACC

	AATGTTGGTGATGCATTAACTGCGCTTGATAACGCAATTAATACTGCGGCAACAACATCTA<br>AATCGACTGTTTCTAATGGTCAGAATATTGTTGTCAGCAAGAGCAAAAATGCAGATGGTT<br>CAGACAACTATGAAGTATCAACAGCAAAAGACTTGACAGTTGATTCTGTCAAAGCGGGT<br>GATACGGTTCTGAATAATGCAGGTATTACAATTGGCAATAACGCAGTTGTATTGAACAAC<br>ACTGGATTAACCATTAGTGGTGGACCAAGTGTTACCTTGGCAGGCATCGATGCAGGCAAT<br>AAAACCATTCAAAATGTTGCGAATGCAGTAAATGCAACAGATGCAGTCAACAAAGGGCA<br>ATTGGACAGCGCAATTAACAATGTGAATAACAATGTAAATGAGCTTGCCAACAACGCTGT<br>TAAATATGACGATGCATCAAAAGATAAGATCACACTTGGTGGTGGGGCAACTGGTACAAC<br>AATCACCAATGTGAAAGATGGTACTGTTGCGCAAGGTTCTAAAGATGCTGTGAATGGCG<br>GTCAATTGTGGAATGTTCAACAACAAGTTGATCAGAACACAACTGATATTAGCAATATCA<br>AAAATGATATTAACAACGGTACTGTTGGTTTGGTTCAACAAGCAGGTAAAGATGCACCAG<br>TGACGGTTGCAAAAGATACTGGCGGTACAACGGTGAATGTCGCTGGAACAGATGGCAAC<br>CGAGTAGTGACAGGTGTTAAGGAAGGTGCAGTGAATGCAACATCTAAAGATGCTGTCAA<br>TGGTAGTCAATTGAATACAACCAACCAAGCGGTAGTCAATTATCTTGGTGGTGGGGCAGG<br>TTATGACAACATTACAGGTAGCTTCACAGCGCCAAGTTATACGGTAGGTGACTCGAAATA<br>CAACAATGTTGGTGGCGCAATTGATGCATTGAATCAAGCAGATCAAGCATTGAATAGCAA<br>AATTGACAATGTCAGTAACAAGTTGGATAACGCATTCCGTATTACCAACAACCGTATTGAT<br>GATGTAGAGAAAAAAGCCAATGCTGGTATTGCCGCTGCGATGGCTCTGGAATCAGCACC<br>ATATGTCCCAGGTAAATATACCTATGCAGCAGGCGCAGCTTACCACGGTGGTGAAAATGC<br>GGTAGGTGTGACTTTACGTAAAACTGCAGACAATGGTCGTTGGTCGATTACAGGCGGTGT<br>AGCTGCAGCGTCTCAAGGCGATGCAAGTGTTCGTATCGGTATCAGCGGTGTGATTGACTA<br>A

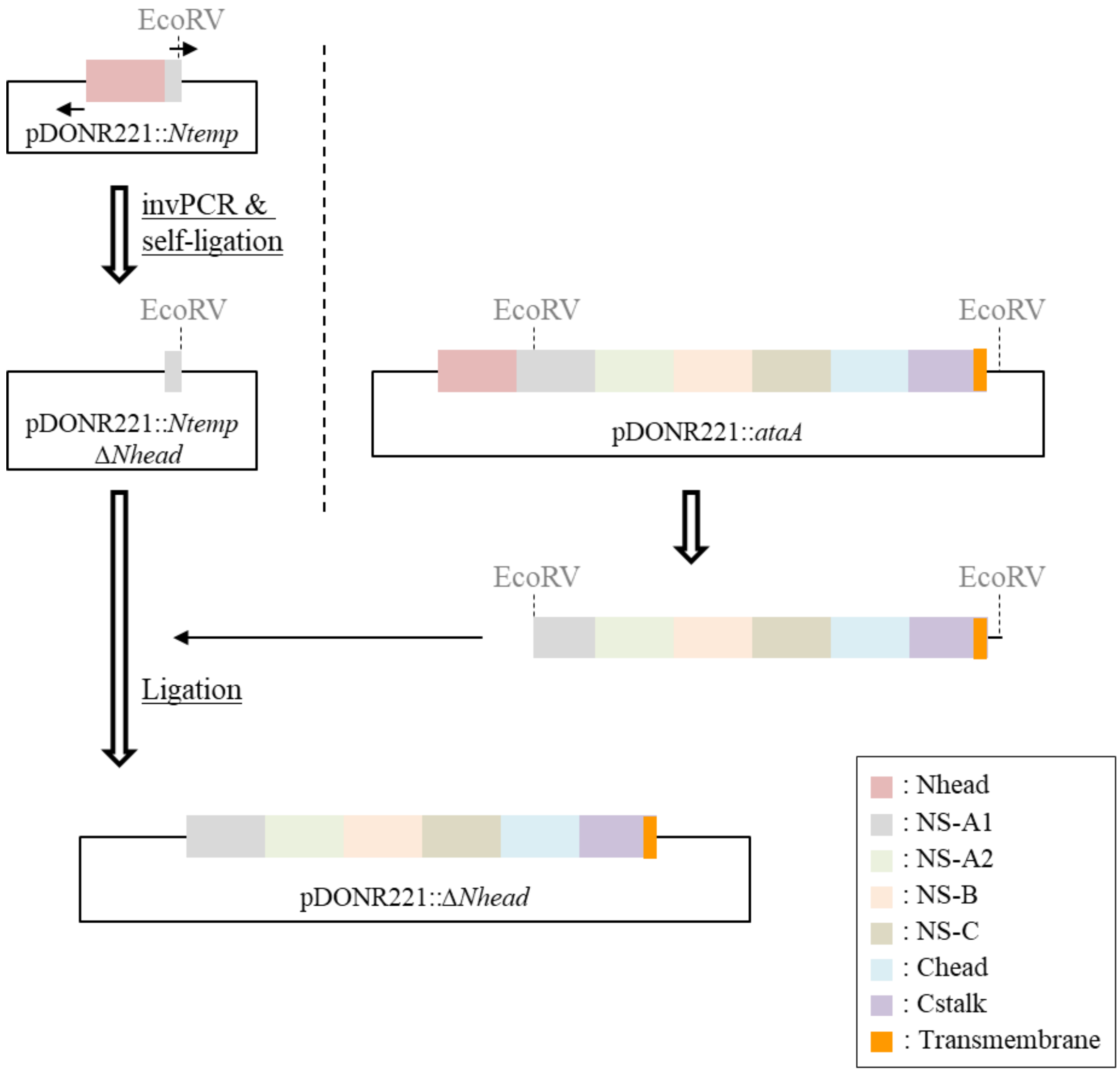

Figure S1. Schematic procedure for the construction of pDONR221::Δ*Nhead*.

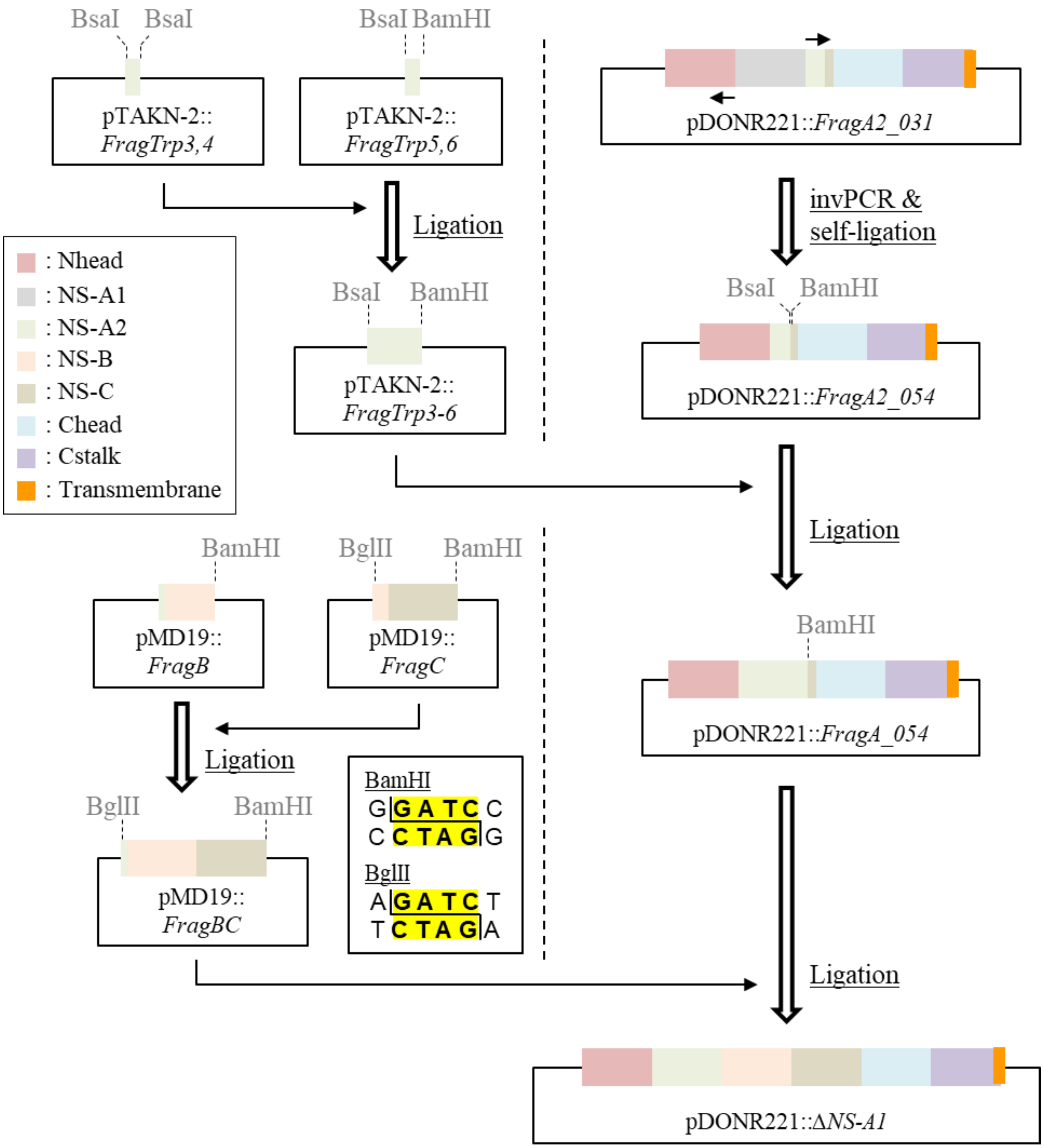

Figure S2. Schematic procedure for the construction of pDONR221::Δ*NS-A1*.

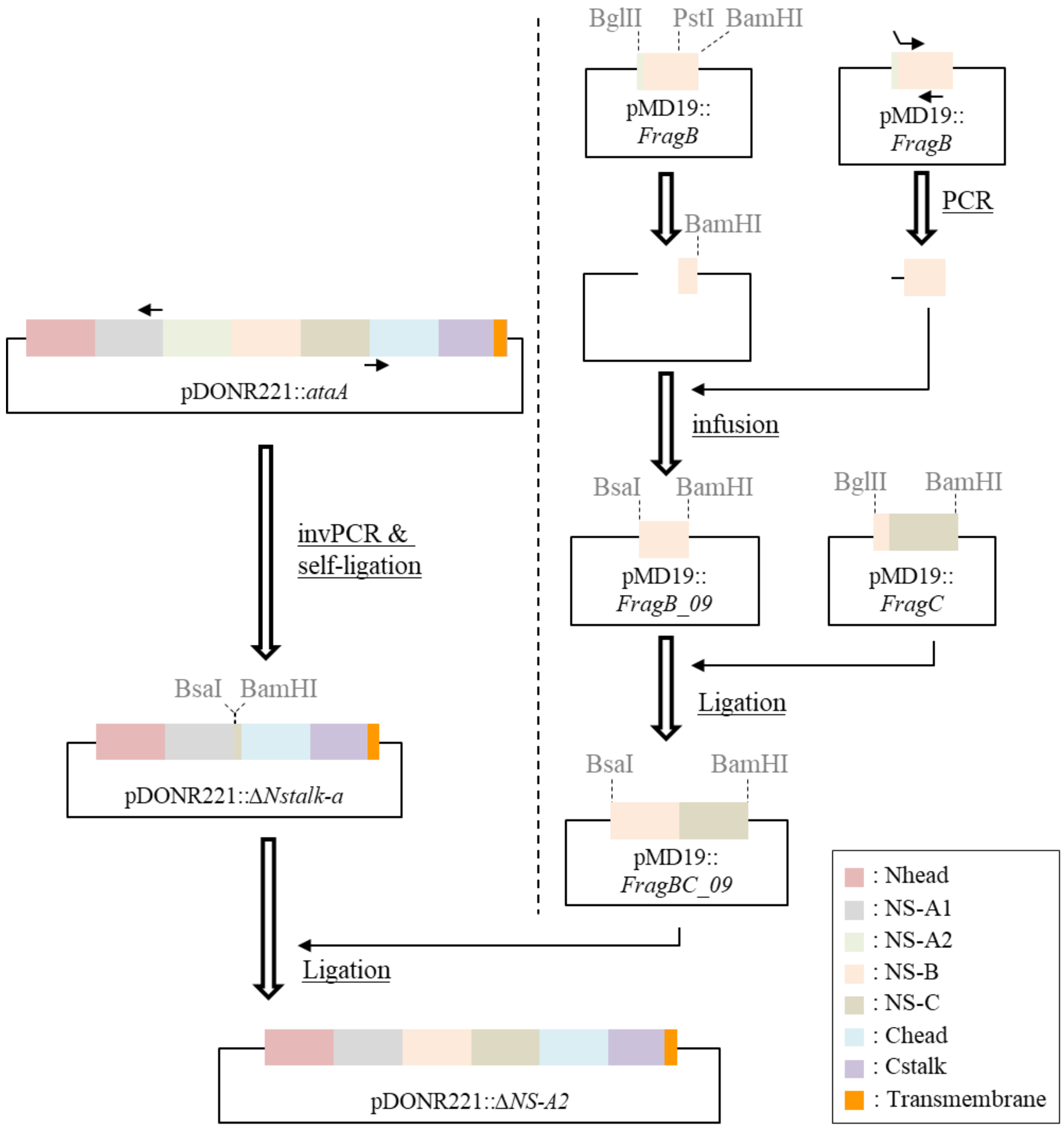

Figure S3. Schematic procedure for the construction of pDONR221::Δ*NS-A2*.

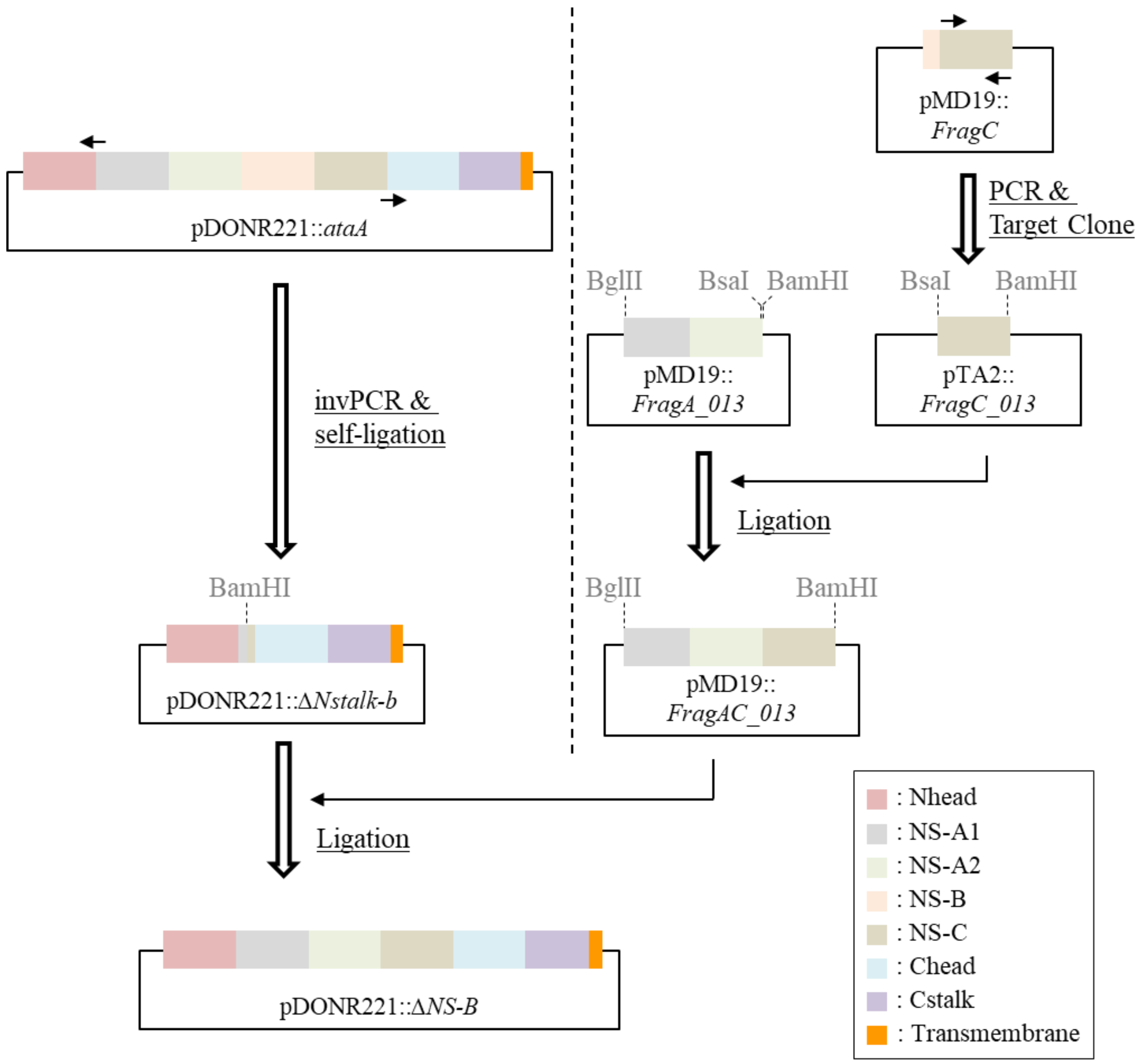

Figure S4. Schematic procedure for the construction of pDONR221::Δ*NS-B*.

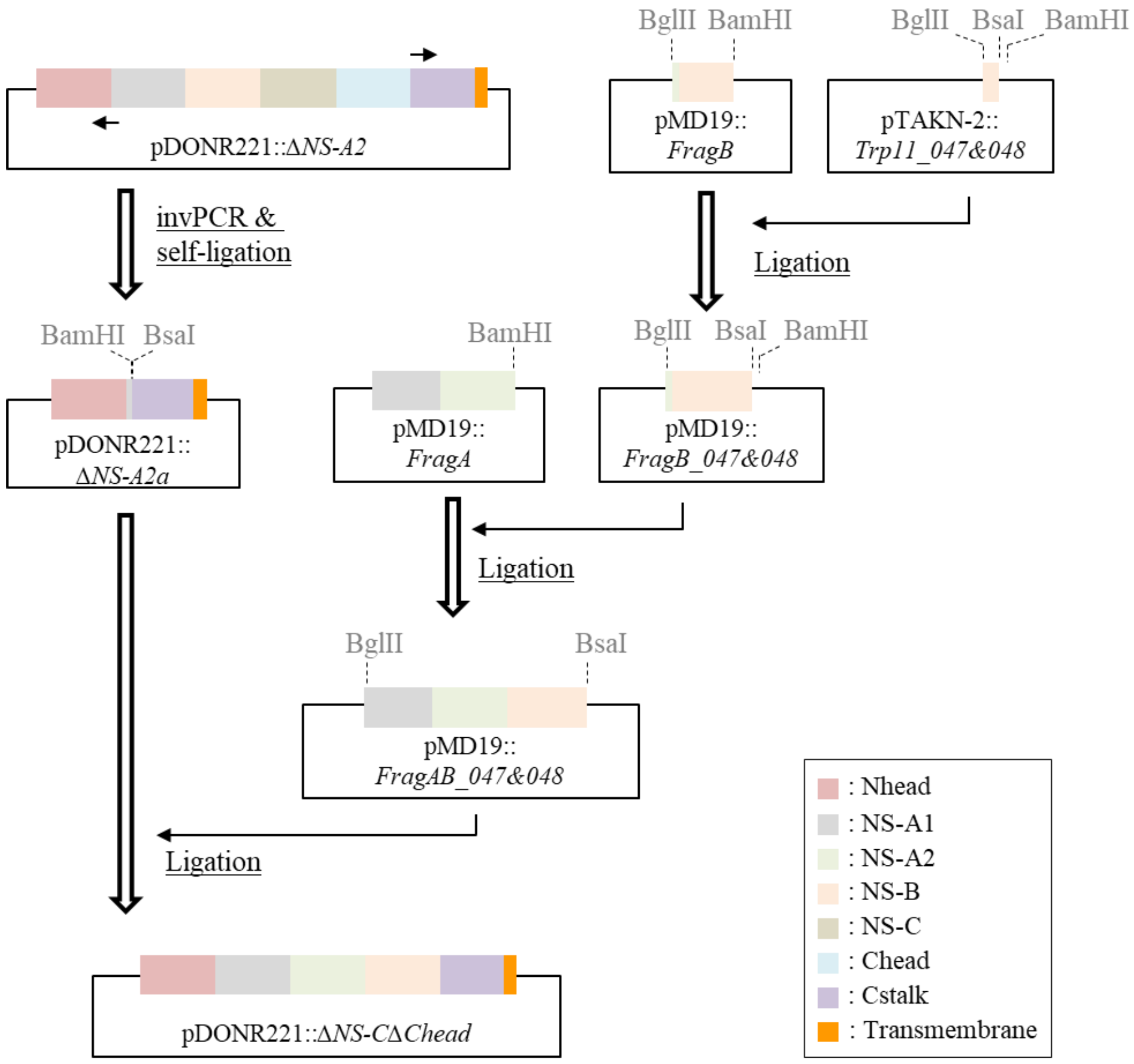

Figure S5. Schematic procedure for the construction of pDONR221::Δ*NS-C*Δ*Chead*.

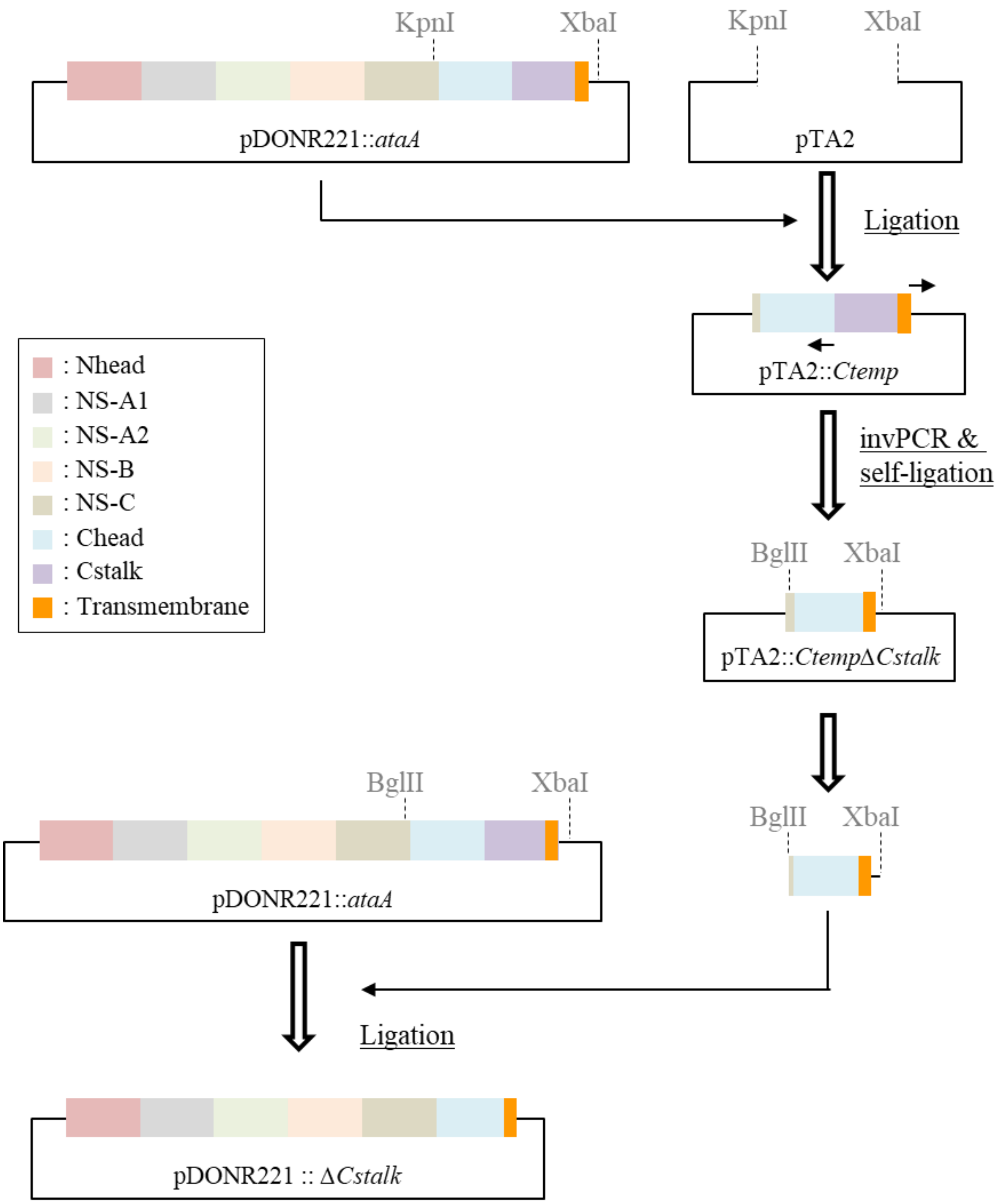

Figure S6. Schematic procedure for the construction of pDONR221::Δ*Cstalk*.

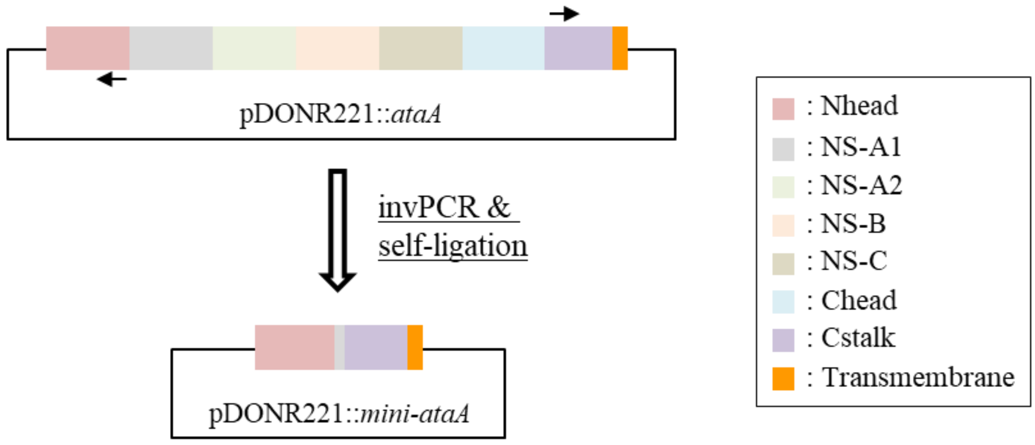

Figure S7. Schematic procedure for the construction of pDONR221::*mini-ataA*.
